# Ex Situ Thermal Preconditioning Modulates Coral Physiology and Enhances Heat Tolerance: A Multispecies Perspective for Active Restoration

**DOI:** 10.1021/acs.est.4c08640

**Published:** 2025-04-25

**Authors:** Erik F. Ferrara, Anna Roik, Franziska Wöhrmann-Zipf, Maren Ziegler

**Affiliations:** † Marine Holobiomics Lab, Department of Animal Ecology and Systematics, Justus Liebig University Giessen, Heinrich-Buff-Ring 26-32 IFZ, 35392 Giessen, Germany; ‡ Helmholtz Institute for Functional Marine Biodiversity at the University of Oldenburg (HIFMB), 26129 Oldenburg, Germany; § Alfred Wegener Institute, Helmholtz Center for Polar and Marine Research, 27570 Bremerhaven, Germany

**Keywords:** stress-hardening, acclimatization, coral bleaching, short-term acute heat stress, thermal tolerance, thermal resilience, trade-off, thermal variability, climate change, ocean warming

## Abstract

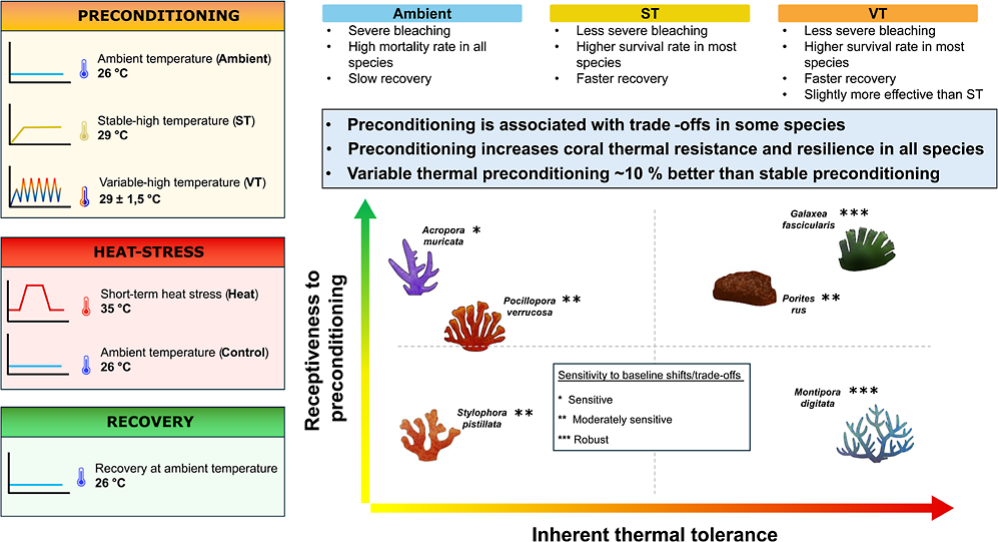

Global warming threatens reef-building corals by challenging their adaptive capacity. Therefore, interventions such as stress-hardening by thermal preconditioning could become crucial for their survival. This study aimed to systematically assess the effects of distinct thermal preconditioning regimes (stable-high at 29 °C, variable-high at 29 ± 1.5 °C, and stable-ambient control at 26 °C) on the baseline physiology and thermal tolerance of six stony coral species (*Galaxea fascicularis*, *Porites rus*, *Acropora muricata*, *Montipora digitata*, *Pocillopora verrucosa*, and *Stylophora pistillata*) to determine commonalities in the stress-hardening responses that transcend species-specific signatures. For this, we quantified changes in photosynthetic efficiency and bleaching intensity before and after a short-term heat stress assay and up to 30 days later. Stress-hardening was successful in all preconditioned corals, with the variable-high regime slightly outperforming the stable-high regime. Preconditioning reduced the heat stress response by up to 90%, yet species differed in receptiveness. It also improved resilience (survival and recovery), and corals with high inherent thermal tolerance recovered better than susceptible species. Notably, both preconditioning regimes affected baseline physiology, exclusively of the branching species, causing tissue paling and decreased photosynthetic efficiency. We conclude that implementing thermal stress-hardening protocols requires consideration of the species-specific receptiveness and potential physiological trade-offs.

## Abstract 2

This study demonstrates the effects of thermal preconditioning on coral heat tolerance and physiological shifts across multiple reef-building species.

## Introduction

Reef-building corals are stenothermal organisms that typically live close to their upper thermal limit,^1,2^ making them highly sensitive to marine heat waves driven by global warming^3,4^ and threatening the existence of tropical reef ecosystems.^5^ These heatwaves cause coral bleaching, the breakdown of the symbiosis between corals and *Symbiodiniaceae* microalgae,^6^ leading to coral starvation and severe health decline.^7−9^

To thrive under environmental changes, corals rely on adaptive evolutionary mechanisms based on genetic variation.^10^ However, the Darwinian adaptation processes of the long-lived corals are too slow to keep up with current rapid environmental changes.^11^ Therefore, acclimatization through phenotypic plasticity and gene expression regulation becomes crucial.^12−17^ Particularly, corals from thermally variable environments, such as reef flats and lagoons, can cope better with thermal extremes than conspecifics living in thermally stable environments.^18−22^ For instance, in *Acropora hyacinthus* inhabiting warmer pools, the expression of heat stress response-related genes is higher than in those from cooler pools, suggesting an inherent readiness for elevated temperatures.^23^ Similar priming effects were observed in *Pocillopora acuta* exposed to short-term sublethal temperatures, which resulted in an increase in thermal tolerance.^24^

Such a response to priming stimuli underpins “stress-hardening approaches”,^12,25−32^ which are based on the deliberate, controlled exposure of organisms to sublethal stimuli (elevated temperatures, increased light intensity, or acidified conditions) to enhance their response to future and more severe stress events.^22^ These approaches may be fundamental for increasing survival of corals in the face of ongoing climate change.^33^

However, the effectiveness of the priming stimulus is likely dose-dependent, and excessively strong stimuli may even be harmful.^28,34,35^ Recent studies reflect these intricacies, documenting results ranging from minor or negligible positive effects^25,36^ to even detrimental outcomes.^37−40^ Further, fine-scale variations in the thermal priming stimulus, such as the diel temperature amplitude or the absolute temperature increase, significantly alter the outcomes of preconditioning regimes within and across coral species.^41−43^ For instance, diel temperature fluctuation with intermediate amplitudes of 2–3 °C can maximize stress tolerance,^34^ whereas larger amplitudes, above 4 °C, may lead to detrimental effects.^35,37,39^ Diel thermal variability generally outperforms stable elevated temperatures,^44^ possibly due to night-time cooling allowing the repair of accumulated stress damage during daytime.^45−47^ Furthermore, the mean temperature of the priming stimulus can affect coral stress-hardening responses. For example, corals exposed to temperatures at least 3 °C above the control (ambient) temperature exhibited enhanced thermal tolerance,^24,35^ whereas a lower increase of 2 °C was insufficient to elicit similar responses.^36^

As species differ in their environmental tolerances, preliminary evidence suggests species-specific receptiveness to thermal priming. For instance, preconditioned *Acropora cervicornis* and *P. acuta* exhibited increased thermal tolerance,^13,14^ whereas other species, such as *Montipora capitata*, showed no improvements.^36,48^ On top of that, local or regional differences linked to environmental gradients or population structure within species add a layer of complexity to these investigations.^25,44,49^

These equivocal results call for a study that systematically compares receptiveness to preconditioning across coral species, as more clarity will be crucial to refine stress-hardening protocols effectively. Furthermore, important knowledge gaps remain, particularly regarding the long-term consequences and potential trade-offs of thermal preconditioning. For instance, exposure to warmer temperatures can improve immediate thermal tolerance through physiological adjustments, such as reduced coral symbiont cell densities.^50,51^ While this shift may aid acclimatization, it likely involves trade-offs, such as reduced growth.^15,52,53^ Furthermore, whether resilience and recovery rates change in stress-hardened corals is unclear, as their long-term fate is rarely monitored. We know that corals from naturally variable habitats recover more rapidly after heat stress compared to those in more stable environments.^21,31,40^ However, to answer the question of whether this increased resilience arises from adaptation or acclimatization requires controlled laboratory experiments.

This study systematically assessed the effects of preconditioning regimes on stony coral species to determine commonalities in the stress-hardening responses that transcend species-specific signatures. The species investigated were: *Galaxea fascicularis* (Linnaeus, 1767), *Porites rus* (Forskål, 1775), *Acropora muricata* (Linnaeus, 1758), *Montipora digitata* (Dana, 1846), *Pocillopora verrucosa* (Ellis & Solander, 1786), and *Stylophora pistillata* (Esper, 1792)—representing four key scleractinian families widely studied in thermal stress research.^54^ Notably, previous research on members of the *Acroporidae* (e.g., *Acropora aspera* and *Acropora millepora*) and *Pocilloporidae* (e.g., *Pocillopora acuta* and *Pocillopora damicornis*) families has demonstrated that short-term preconditioning can alter their physiology and enhance their thermal stress tolerance during simulated long-term heat waves.^14,20,55,56^ In contrast, other *Acroporidae* (e.g., *Montipora capitata*) and *Poritidae* (e.g., *Porites lobata*) members were not receptive to preconditioning.^36,37^ Corals were preconditioned with three distinct thermal regimes—stable-high (29 °C), variable-high (29 ± 1.5 °C), and stable-ambient (26 °C)—for 24 days and thereafter exposed to an acute heat stress test. Physiological parameters were monitored throughout the experiment to evaluate how preconditioning regimes affected (1) baseline physiology after preconditioning, (2) thermal tolerance to acute heat stress, and (3) long-term resilience (survival and recovery 30 days poststress). Ultimately, we aimed to (4) identify the universally most effective preconditioning regime for all coral species.

## Materials and Methods

### Experimental Overview and Coral Species

**Figure 1:**
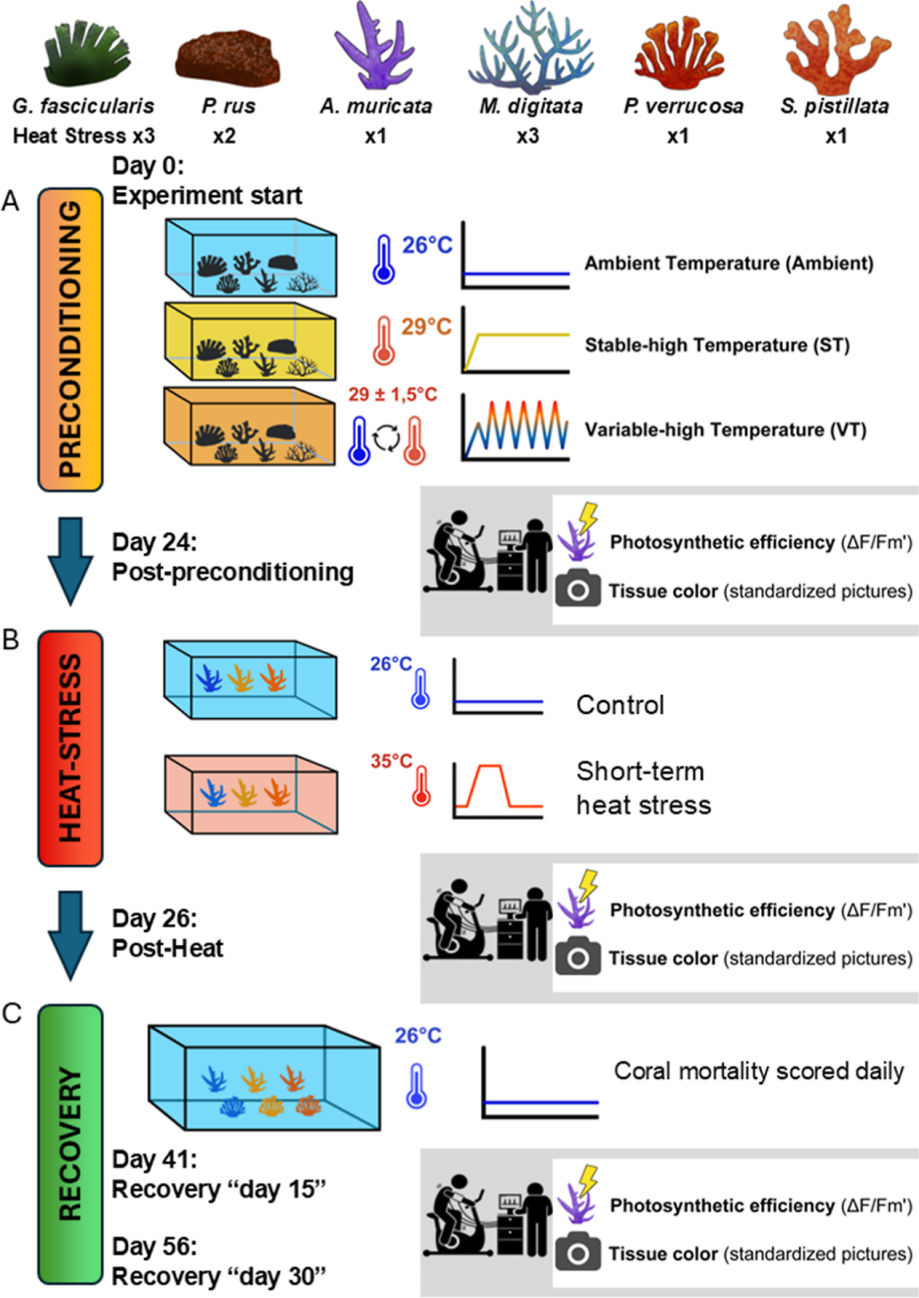
Study design and thermal stress-hardening regimes. Six stony coral species were exposed to three thermal preconditioning regimes (A), followed by short-term heat stress assays (B) to assess the changes in thermal tolerance (each species was exposed to a different number of heat stress cycles as necessary). Photosynthetic efficiency (effective quantum yield of photosystem II) and tissue color intensity (from standardized pictures) were measured as coral response variables at two time points: after the preconditioning phase (“post-preconditioning”) and after acute heat stress (“post-heat”). Corals were placed in a common tank (C) and survival was monitored over 30 days. Response variables were measured on days 15 and 30 after heat stress exposure.

Six stony coral species from four families, representing common reef-builders in the Indo-Pacific, were selected to investigate the effects of three thermal regimes on baseline physiology, heat stress tolerance, and long-term resilience. Corals with two distinct growth forms were included, with *G. fascicularis* and *P. rus* as massive species, and *A. muricata*, *M. digitata*, *P. verrucosa*, and *S. pistillata* as branching species. These corals were collected from different reef locations worldwide (Table S1) and cultivated at the *Ocean2100* coral aquarium facility at the Justus Liebig University Giessen, Germany, for 2–6 years before the experiment. In July 2021, 432 coral fragments were produced from four to eight colonies per species (Table S1). Each colony was cut into 12 fragments of ∼3–4 cm in length and maintained in 265 L tanks at 26 °C for at least 10 weeks before the start of the experiment. Each tank was connected to a recirculating artificial seawater system with water exchange of 0.7 L/min, water flow of 3–6 cm/s, and a 10:14 light/dark photoperiod with a light intensity of 250 ± 30 μmol photons m^–2^ s^–1^ (measured by Apogee Lightmeter, Model MQ-510). The water temperature of each tank was feedback-controlled (GHL Temp Sensor digital, ProfiLux 3 and 4, GHL Advanced Technology GmbH, Germany, and Schego Heater 300 and 600 W, Schemel & Goetz GmbH, Germany) and recorded every 10 min (HOBO MX Pendant Temp, MX2201, Onset, USA). Corals were fed with copepods 3 days per week (Calanoide Copepoden, Zooschatz, Germany), except during acute heat assays when no food was provided. Due to logistic reasons, the experiment was conducted in three consecutive runs using two coral species at a time: (1) *A. muricata* and *M. digitata*, (2) *P. verrucosa* and *S. pistillata*, (3) *G. fascicularis* and *P. rus*. The preconditioning phase for the experimental runs started on 23.11.2021, 11.01.2022, and 04.02.2022, respectively. Each experiment consisted of three phases: the preconditioning phase, the acute heat stress test phase, and the recovery phase (Figure 1).

### Preconditioning Phase

During the preconditioning phase, corals were exposed to three thermal regimes for 24 days, including a stable-high temperature (ST), a variable-high temperature (VT), and a stable-ambient temperature (Ambient) as control regime (Figure 1A). Twelve fragments per colony were evenly distributed among preconditioning treatment tanks (four fragments per preconditioning regime).

The stable-ambient regime was held at 26 ± 0.5 °C, corresponding to the facility baseline temperature. In the stable-high temperature regime, the temperature was increased from 26 to 29 °C at 1 °C day^–1^ and held constant for 18 days (Figure 1 A). The variable-high temperature regime underwent the same temperature increase to 29 °C, but then a diel fluctuation with a 3 °C amplitude around 29 °C was imposed for 18 days (Figure 1A). Subsequently, the temperatures in both ST and VT regimes were decreased back to 26 °C within a day and maintained constant for two more days, resulting in a preconditioning phase of 24 days.

To quantify cumulative heat exposure, we calculated degree heating weeks (DHW) for each experimental treatment. The cumulative heat exposure in our preconditioning treatments was calculated by summing all daily temperature anomalies above the bleaching threshold of 27 °C (1 °C above the maximum monthly mean, MMM = 26 °C). Following the temperature ramp-up, corals were exposed to two distinct thermal regimes for 18 days, and both regimes resulted in the same total cumulative heat exposure of 7.71 DHW. Corals in the ST treatment were maintained at a constant 29 °C for 18 days. For each day, the temperature anomaly was 3 °C above MMM (29 °C – 26 °C = 3 °C). The cumulative heat exposure was, therefore, 3 °C × 18 days = 54 °C·days, corresponding to 7.71 DHW (54 °C·days ÷ 7 days). The VT treatment followed a diel cycle, reaching 30.5 °C during the daytime and 27.5 °C during the nighttime. The daytime anomaly was (30.5 °C – 26 °C) = 4.5 °C for 12 h, and the nighttime anomaly was (27.5 °C – 26 °C) = 1.5 °C for 12 h of every day. Over 24 h, the combined daily anomaly averaged to ((4.5 °C × 12 h) + (1.5 °C × 12 h)) ÷ 24 h = 3 °C. The total cumulative heat exposure over 18 days thus also corresponds to 7.71 DHW.

### Heat Stress Phase

Heat stress assays were conducted after the preconditioning phase (Figure 1B). The assays were set up using 12 × 40 L tanks, each equipped with a current pump (easyStream pro ES-28, AquaLight GmbH, Bramsche/Lappenstuhl, Germany) and 65 μm mesh inflow filters to prevent the intrusion of particles. The heat stress assays were run at a light intensity of ∼120 μmol photons m^–2^ s^–1^ (white and blue sunaECO LED, AquaRay by Tropical Marine Centre, United Kingdom). For each run, a total of 144 fragments (two species, 12 colonies) were distributed among 12 tanks (six heat and six control), with 12 fragments per tank. Each tank contained three fragments per colony, one from each preconditioning regime of the same species. The control tanks were maintained at 26 °C. In the heat treatment, the temperature was rapidly increased from 26 to 35 °C over 3 h, held at 35 °C for 3 h, and then returned to 26 °C within 2 h. Each heat stress assay began at 15:00 h, and following Voolstra et al.,^54^ coral responses were assessed 18 h later, at 9:00 am of the following day. Because a meaningful assessment of the heat stress responses critically depends on reaching a point of differential bleaching between preconditioning treatments, coral species were exposed to multiple heat stress cycles depending on their inherent heat stress tolerance following Doering et al.^57^ This was achieved by applying the same heat-stress schedule twice for *P. rus* and three times for *G. fascicularis* and *M. digitata*.

### Recovery Phase

To understand how priming trade-offs and short-term heat stress resistance were related to long-term resilience, corals were monitored for 30 days following the short-term heat stress assays. For this, half of the coral fragments were transferred to a recovery aquarium at 26 °C for 30 days (Figure 1C), while the other half was frozen for further analyses.^55^ This division resulted in a 50% reduction in sample size for each species during the recovery period. As the 30 day window can be the most critical period during which coral recovery or decline is most evident,^58,59^ mortality was recorded daily, with fragments deemed dead when no tissue remained on the skeleton. Recovery was assessed by measuring photosynthetic efficiency and changes in tissue color (bleaching) at 15 and 30 days.

### Coral Stress Response Measurements

Physiological response variables were measured for each fragment at the end of the preconditioning and heat stress phases, as well as during the recovery period on day 15 and after the recovery period on day 30. Since the post-preconditioning measurements of the ambient treatment reflect the physiological status of corals, both before and after preconditioning, as such, providing a comprehensive overview of the baseline parameters of corals, we did not assess physiological measurements before the preconditioning phase. Heat stress phase data presented here always refer to the measurements taken after the last heat stress cycle of each species.

We measured the effective quantum yield of photosystem II (YII) as a proxy of Symbiodiniaceae health using a pulse-amplitude modulation (PAM) fluorometer equipped with a clear plastic tube at the tip of the fiber optic cable to keep a stable distance to the coral surface at a 45° angle (PAM-2500 Portable Chlorophyll Fluorometer, Heinz Walz GmbH, Germany). Tissue color intensity was assessed as a proxy for coral bleaching, as it has been shown to scale linearly with symbiont density.^55^ Lighter colors indicated a decrease in symbiont density and, thus, a stronger stress response or bleaching severity. Tissue color intensity was obtained from standardized pictures of each coral fragment documented with a digital SLR camera (Nikon D7000) in an evenly illuminated macro photo studio (80 × 80 × 80 cm, Life of Photo). Fragments were placed on a black background with the larger side facing the camera, next to a reference color card for white balance (ColorChecker Passport Photo 2, Calibrite, US). First, the background was removed from each image in Adobe Photoshop 2020. Cropped images were then analyzed with a Python script,^56^ extracting the gray channel value for each pixel to create a color intensity histogram. The mean value was used to estimate the tissue color intensity. Bleaching severity was assessed from tissue color intensity on a scale of 0 to 255, in which 0 corresponds to white and 255 corresponds to black. While 0 corresponds to pure white, the lowest value reached in completely bleached fragments was 30. Photosynthetic efficiency and tissue color intensity of dead coral fragments during the recovery phase were manually set to 0 and 30, respectively.

### Statistical Analyses

All analyses were performed in the *R* statistical environment (version 4.2.3; R Core Team, 2022) with the package *ggplot2* for visualization,^60^*dabestR* v0.3.0 for effect size calculations,^61^*lme4* v 1.1–35.2 for linear mixed effect models,^62^ and *car* for ANOVAs.^63^ We conducted all analyses separately for each physiological parameter and coral species. Shapiro–Wilk tests were used to test the normality of the data. Bartlett’s test and the Breusch-Pagan test were used to confirm homogeneity of variances between groups and constant variance of residuals, respectively.

First, the effects of the thermal preconditioning regimes on baseline physiology of the corals were determined. For this, tissue color intensity and effective photosynthetic yield measured at the end of the preconditioning phase were analyzed separately per species. Differences in physiology between the ST and VT preconditioning regimes compared to the Ambient were calculated as pairwise Hedges’ *g* effect sizes using raw data. Then, differences were tested with linear mixed effect models applying thermal regimes (ST, 29 °C vs VT, 29 ± 1.5 °C vs Ambient, 26 °C) as fixed factor and coral colony genotype as random factor. Normality and homoscedasticity for each model were checked, and where assumptions were violated, data were transformed to improve model performance. ANOVAs were used to compute *F* statistics of the linear mixed effect models.

Next, the effects of short-term heat stress exposure on the physiology of corals from the three thermal regimes were assessed. Physiological values measured after preconditioning were used as a reference and compared against those measured after heat stress within each preconditioning group with nonparametric Wilcoxon tests. A significant difference indicated the decline in physiological performance due to heat stress exposure. To test whether the preconditioning regime affected the intensity of the heat stress response, we calculated Hedges’ *g* effect sizes within each preconditioning regime on raw data. Then, for each physiological variable for each coral fragment, a heat stress response metric was generated by calculating Δ-values (“post-heat” minus “post-preconditioning” measurements) indicative of the magnitude of the physiological decline due to heat stress. Statistical models compared the differences of Δ-values as a proxy for the stress response between the three preconditioning groups using a linear mixed effect model with coral colony genotype and tank number as random factors. ANOVAs were used to compute *F* statistics of the linear mixed effect models.

Last, we evaluated whether the effect of heat stress on physiology was still detectable in the three thermal regimes 30 days after heat exposure. For this, we calculated the effect size as Hedge’s g between control samples not exposed to heat stress and the heat-exposed samples within each preconditioning regime, followed by two-sided permutation *t* tests. Survival was scored throughout (1 = alive, 0 = dead) and analyzed with the *R* package *survival*(64) and visualized using Kaplan–Meier plots. A heat map of all effect sizes of stress responses, recovery, and survival rates for each coral species was created to summarize and integrate all results into one figure using *ggplot2*.

## Results

### Effects of Thermal Preconditioning on Baseline Physiology

**Figure 2:**
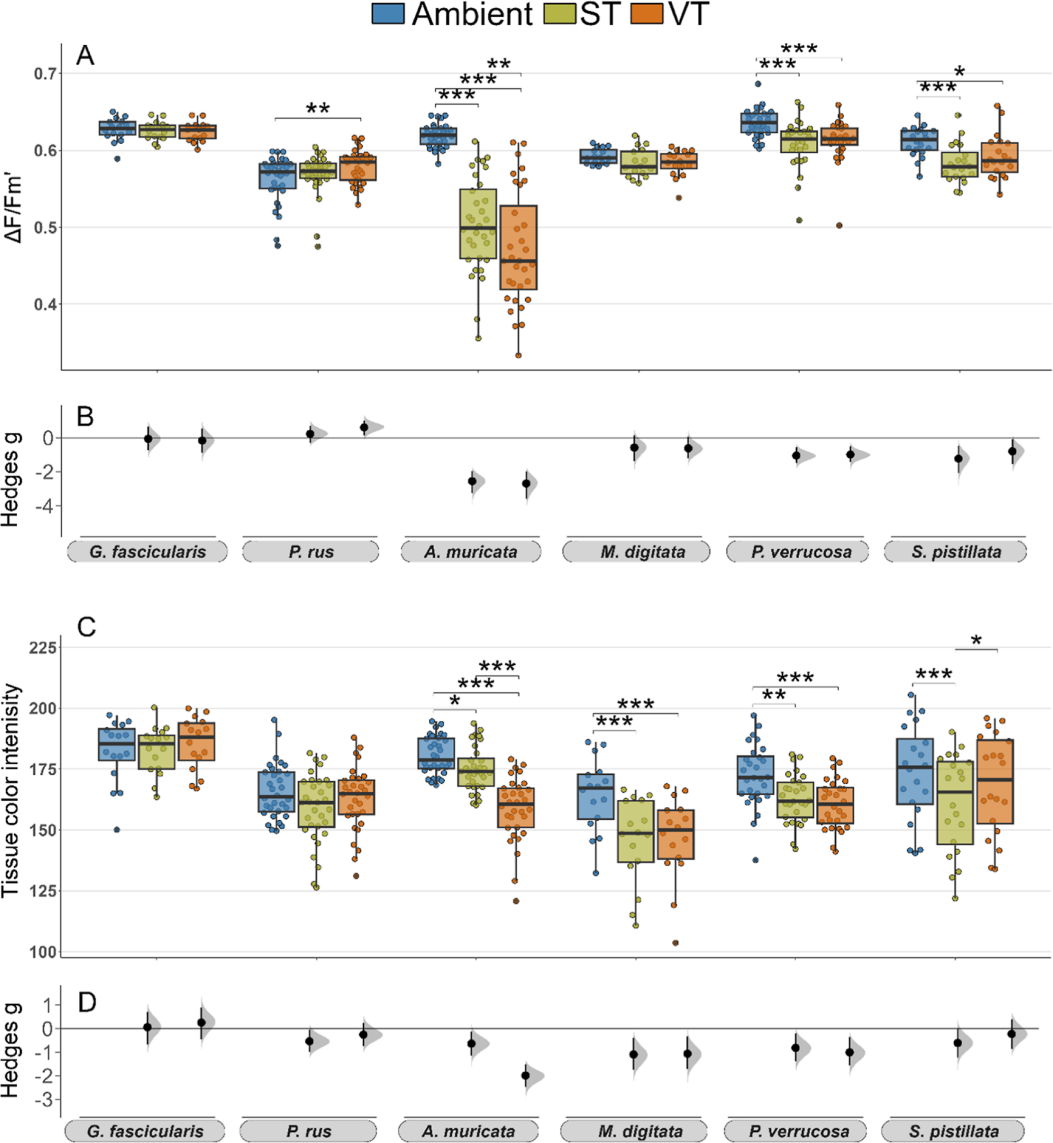
Immediate changes in coral baseline physiology following thermal preconditioning. The changes in effective quantum yield (Δ*F*/*F*_m_′) (A) and tissue color intensity (C) in response to preconditioning thermal regimes are shown as boxplots, pairwise differences between the stable-high (ST) and variable-high (VT) treatments compared to the stable-ambient (ambient) treatment are shown as Hedges’ *g* effect sizes including the 95% CIs (B,D). Data in (A,C) are displayed as boxplots with raw data points; lines indicate medians, boxes indicate the first and third quartile, and whiskers indicate ±1.5 IQR. Connecting lines between boxes indicate significant differences between preconditioning regimes (*p* < 0.001***, *p* <0.01**, *p* <0.05* from linear mixed effect models). Sample sizes per species: *G. fascicularis* = 16, *P. rus* = 32, *A. muricata* = 32, *M. digitata* = 16, *P. verrucosa* = 28, *S. pistillata* = 20.

Overall, photosynthetic efficiency and tissue color intensity declined in response to both high-temperature preconditioning regimes (Figure 2). Four of the six tested coral species preconditioned under stable-high temperature (ST, 29 °C) and variable-high temperature (VT, 29 ± 1.5 °C) had significantly lower photosynthetic efficiency and/or tissue color intensity compared to corals in the stable-ambient temperature regime (Ambient, 26 °C). Consequently, we classified the six coral species into three main groups based on the magnitude of physiological shifts observed after thermal preconditioning: (1) “robust” for species with stable baseline physiology; (2) “moderately sensitive” for species exhibiting a moderate shift; and (3) “sensitive” for species with large declines in baseline physiology (Table 1).

**Table 1:** Experimental Classification of Thermal Properties of the Six Studied Coral Specie^a^

species	no. of heat stress cycles	sensitivity to baseline shifts	receptiveness to stress hardening increased heat stress tolerance	recovery ranking
*G. fascicularis*	3	robust	receptive	+++
*M. digitata*	3	moderately sensitive	not receptive	+++
*P. rus*	2	robust	moderately receptive	++
*P. verrucosa*	1	moderately sensitive	moderately receptive	+
*A. muricata*	1	sensitive	receptive	–
*S. pistillata*	1	moderately sensitive	not receptive	–

a The number of heat stress cycles needed to reach differential bleaching is shown. Coral species were classified based on their sensitivity to baseline physiological shifts through preconditioning as follows: “robust” when their physiological parameters remained stable, “moderately sensitive” when their parameters exhibited a small but significant decline, and “sensitive” when their parameters showed a large decline, close to a stress response. The receptiveness of each species to stress hardening through preconditioning (i.e., increasing heat stress tolerance) was scored as “receptive” when the heat stress response of preconditioned corals was significantly lower than that of the ambient group, “moderately receptive” when they exhibited minor mitigation of the stress response and/or inconsistent responses, and “not receptive” when the heat stress response was similar across all preconditioning groups. Recovery was ranked according to the following criteria: +++ when all stress-hardened corals survived and recovered at a higher rate than the ambient group, ++ when more than 50% of stress-hardened corals survived and recovered at a higher rate than the Ambient group, + when less than 50% of stress-hardened corals survived and only partially recovered, despite responding better than the ambient group, – when none of the stress-hardened corals survived after 30 days.

We classified *G. fascicularis* and *P. rus* as “robust” species as their photosynthetic efficiency and tissue color intensity remained stable in response to the thermal preconditioning treatments (Figure 2, Tables 1 and S2). Specifically, the treatments had no significant effect on the baseline physiology in *G. fascicularis* (*p* > 0.05) and only minor effects in *P. rus*, where photosynthetic efficiency was slightly increased in the VT treatment compared to the ambient treatment (*g* = 0.61, *p* < 0.01; Figure 2A).

We classified *M. digitata*, *P. verrucosa*, and *S. pistillata* as “moderately sensitive” based on a small but significant decline in their physiological performance in response to thermal preconditioning (Figure 2, Tables 1 and S2). Particularly, photosynthetic efficiency significantly decreased by ∼5% in *P. verrucosa* (ST *g* = −1.04, *p* < 0.001; VT *g* = −0.98, *p* < 0.001) and *S. pistillata* (ST *g* = −1.22, *p* < 0.001; VT *g* = −0.79, *p* < 0.05) compared to the ambient treatment. In contrast, it remained stable in all preconditioning groups for *M. digitata* (*p* > 0.05; Figure 2A,B, Table S2). Tissue color intensity significantly decreased *M. digitata* (ST *g* = −1.09, *p* < 0.001; VT *g* = −0.62, *p* < 0.001) and *P. verrucosa* (ST *g* = −0.81, *p* < 0.01; VT *g* = −1.00, *p* < 0.001) fragments on average by 11 and 7%, respectively. Tissue color intensity was significantly reduced in *S. pistillata* ST treatment compared to the Ambient treatment (*g* = −0.60, *p* < 0.001), and it was also statistically different from the VT treatment (*p* < 0.05, Figure 2C,D, Table S2).

We ranked *A. muricata* as a “sensitive” species based on the comparably large effects of thermal preconditioning on its baseline physiology, which were approximately twice as large as that of the “moderately sensitive” coral species (Table 1). Photosynthetic efficiency decreased by roughly 20% in ST (*g* = −2.55, *p* < 0.001) and VT corals (*g* = −2.68, *p* < 0.001), reaching levels of Δ*F*/*F*_m_′ below 0.5. Moreover, the decrease in photosynthetic efficiency of VT corals was significantly larger than in ST corals (*p* < 0.01). Similarly, the decrease in tissue color intensity was significantly larger in the VT than in the ST group (*p* < 0.001), which both decreased by 3 and 12% on average compared to the Ambient group, respectively (ST *g* = −0.63, *p* < 0.05, VT *g* = −1.99, *p* < 0.001; Figure 2, Table S2). Nonetheless, preconditioned *A. muricata* fragments appeared slightly paler, but visually healthy (Figure S1b). Overall, both preconditioning treatments induced similar baseline physiological changes in all coral species except for *A. muricata* and *S. pistillata*, where the physiological response of VT corals was significantly different from ST corals.

### Effects of Thermal Preconditioning on Coral Thermal Tolerance

**Figure 3:**
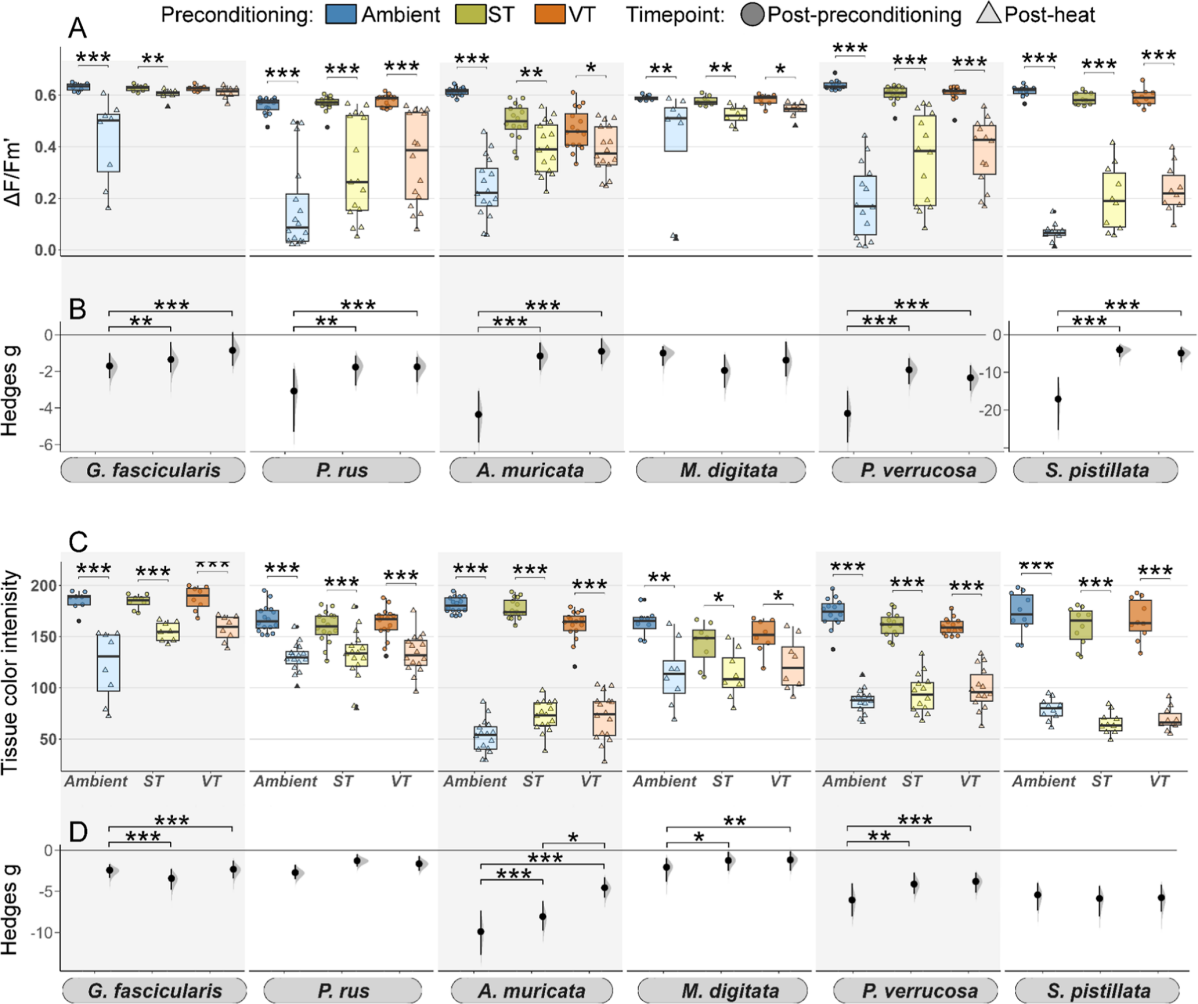
Coral thermal tolerance assessed in heat stress assays following the thermal preconditioning treatments. The decline in effective quantum yield (Δ*F*/*F*_m_′) (A) and tissue color intensity (C) in response to heat stress within each preconditioning group is shown as boxplots, comparing “post-heat” (lighter color) and “post-preconditioning” (darker color) values. Pairwise differences between time points within each preconditioning regime are shown as Cumming estimation plots on Hedges’ *g* effect sizes, including the 95% CIs (B,D). Data in (A,C) are displayed as boxplots with raw data points. Connecting lines between boxes indicate significant differences between time points (*p* < 0.001***, *p* < 0.01**, *p* < 0.05* from Kruskal–Wallis and post hoc Wilcoxon test). Significant differences in heat stress responses between ST and VT compared to the Ambient group are calculated based on the paired difference between “post-heat” and “post-preconditioning” time points and indicated by connecting lines (*p* < 0.001***, *p* < 0.01**, *p* < 0.05* from the linear mixed effect model). Sample size per species: *G. fascicularis* = 8, *P. rus* = 16, *A. muricata* = 16, *M. digitata* = 8, *P. verrucosa* = 14, *S. pistillata* = 10.

Photosynthetic efficiency and tissue color intensity significantly decreased in all coral species in response to the acute heat stress (Figure 3). However, the heat stress response was consistently more severe in the Ambient corals than those from the ST and VT treatments and differences in photosynthetic efficiency were more intense than in tissue color (bleaching). We classified the coral species into three groups based on their receptiveness to stress-hardening treatments (ST and VT). We classified coral species as (1) “receptive” to stress-hardening when their heat stress response was significantly smaller in the ST and VT preconditioning groups compared to the ambient group; (2) as “moderately receptive” when they showed only minor mitigation in stress response and/or responses were inconsistent; (3) and as “not receptive” when the heat stress response was similar across all preconditioning groups (Table 1).

*G. fascicularis*and *A. muricata* were classified as “receptive” to the stress-hardening effect of thermal preconditioning, as their heat stress response was reduced in both response variables (Figure 3, Table 1). In *G. fascicularis* and *A. muricata* the declines in photosynthetic efficiency in the ST and VT groups were significantly reduced by ∼90 and 80% respectively, compared to the Ambient group (ST *p* < 0.01; VT *p* < 0.001; Figure 3A,B, Tables S3, S5 and S6). Additionally, ST and VT corals bleached significantly less (*p* < 0.001; Figure 3C,D, Tables S4–S6). In *G. fascicularis*, bleaching of ST and VT corals was half as severe as in the Ambient group (*p* < 0.001; Figure 3C). While *A. muricata* bleached more severely across all three regimes, the decline in tissue color was significantly smaller in the ST and VT groups compared to the Ambient (*p* < 0.001; Figure 3C,D). Furthermore, the bleaching severity of VT *A. muricata* fragments was significantly lower than those from the ST group (*p* < 0.5; Figure 3D).

*P. rus* and *P. verrucosa* were classified as “moderately receptive” to thermal preconditioning. In these species, the large decrease in photosynthetic efficiency in the ambient group was mitigated in the ST and VT corals (ST *p* < 0.01; VT *p* < 0.001) while bleaching severity was similar across preconditioning treatments (Figure 3, Table 1). The decline in photosynthetic efficiency of ST and VT corals was ∼57% smaller than Ambient *P. rus* and *P. verrucosa* fragments (Figure 3A,B, Tables S3, S5 and S6). The severity of coral bleaching in response to heat stress was more homogeneous across all preconditioning treatments in these “moderately receptive” species than in the “receptive” species (Figure 3C,D, Tables S4–S6). In *P. rus*, bleaching severity was similar across all preconditioning regimes (*p* > 0.05), while in *P. verrucosa*, ST and VT fragments bleached 30% less than in the ambient treatment (ST *p* < 0.01; VT *p* < 0.001; Figure 3C,D).

Despite the strong difference in inherent heat tolerance between *M. digitata* and *S. pistillata*, both corals were classified as “not receptive” to thermal preconditioning. In both corals, none of the treatments had any relevant effect on the stress responses, however, the species-specific responses were very different (Figure 3, Table 1). In *M. digitata*, the decrease in photosynthetic efficiency after heat stress was minor and the difference between preconditioning treatments was not significant (*p* > 0.05; Figure 3A,B, Tables S3, S5 and S6). Additionally, bleaching in *M. digitata* was overall low, with slightly less bleaching in the ST and VT groups compared to the ambient (*p* > 0.01; Figure 3C,D). In contrast, photosynthetic efficiency severely decreased across all preconditioning treatments in *S. pistillata*, as illustrated by the largest effect sizes of the heat stress treatment across all species (Figure 3B) and, although ST and VT preconditioning had a positive effect compared to the Ambient regime (*p* < 0.001), photosynthetic efficiency was extremely low in all groups after heat treatment (i.e., Δ*F*/*F*_m_′ ∼0.2, Figure 3A,B, Tables S4–S6). Moreover, all fragments bleached severely with no difference between the preconditioning treatments (*p* > 0.05; Figure 3C,D). Overall, all species demonstrated a similar receptiveness to both ST and VT preconditioning treatments at large, regardless of the species-specific level of stress mitigation.

### Effects of Thermal Preconditioning on Coral Survival and Recovery

**Figure 4:**
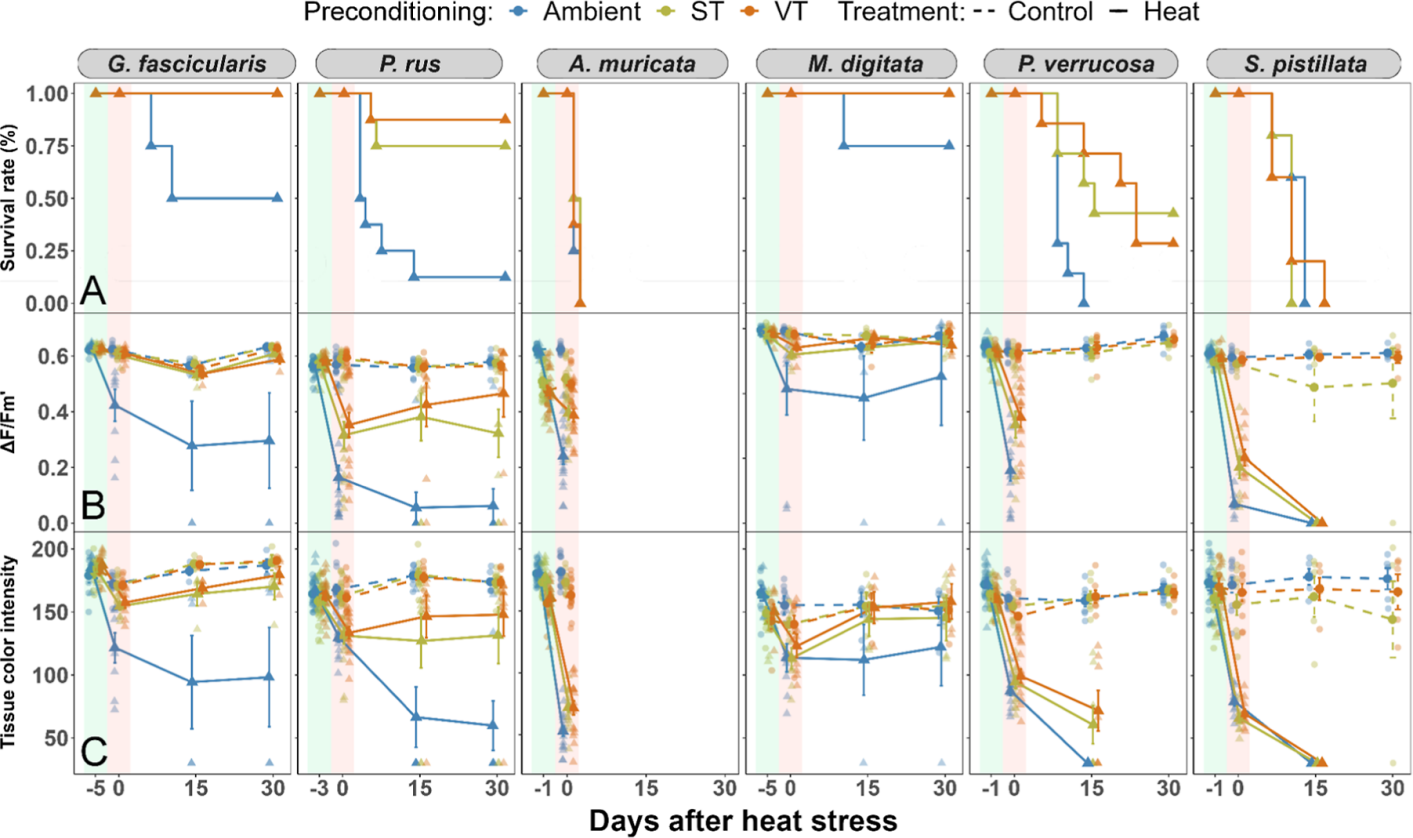
Coral survival rates and recovery following the heat stress assays. Kaplan–Meier plots show changes in coral survival rates under ST, VT, and Ambient preconditioning regimes (A). The trajectories of effective quantum yield (Δ*F*/*F*_m_′) (B) and tissue color intensity after the heat stress assays (C) for corals from the same preconditioning regimes reflect on the recovery of corals. Lines connect mean values (solid lines for heat treatment, dashed lines for control treatment), and raw data points are included. These recovery lines are based on data from a constant sample size within each “population” of each preconditioning regime. The effective quantum yield of dead coral fragments was recorded as 0, while tissue color intensity was scored as 30 (the lowest score observed after the heat stress assay). Physiological parameters of alive *P. verrucosa* fragments were not scored due to uncertainties introduced by algal growth on necrotic tissue, which started dominating the corals between day 0 and 15. Sample size per species: *G. fascicularis* = 4, *P. rus* = 8, *A. muricata* = 8, *M. digitata* = 4, *P. verrucosa* = 7, *S. pistillata* = 5.

Coral survival, photosynthetic efficiency, and tissue color were monitored for 30 days after the acute heat stress to evaluate the effect of thermal preconditioning regimes on coral recovery trajectories (Figure 4). Overall, coral survival was consistently lower in the Ambient group than in the ST and VT groups (Figure 4A). Specifically, in the two species with highest survival rates throughout the recovery period (i.e., *G. fascicularis* and *M. digitata*) all ST and VT fragments survived the heat treatment, while the survival rate was only 50 and 75% in the Ambient treatment, respectively (Figure 4A). Moreover, photosynthetic efficiency and tissue color of *G. fascicularis* fragments from the ambient preconditioning treatment did not fully recover within the 30 days after heat stress (*p* < 0.05; Figure 4B,C). In contrast, both metrics recovered to prebleaching levels in VT fragments (*p* > 0.05; Figure 4B,C). In *M. digitata*, photosynthetic efficiency and tissue color of fragments recovered in all preconditioning groups after 30 days, with slightly slower recovery in corals from the ambient preconditioning group (*p* > 0.05; Tables S4 and S6).

Importantly, corals with an inherently higher thermal tolerance exhibited higher survival and recovery than the less heat-stress tolerant species (Table 1). After *G. fascicularis* and *M. digitata*, *P. rus* was the second most heat-stress tolerant species, and it also benefited from the preconditioning, as 75 and 87% of ST and VT corals survived after heat stress, respectively, compared to 12% of the ambient treatment (Figure 4A, Table 1). Recovery of photosynthetic efficiency and tissue color was highest in the VT group, where both metrics regained roughly half of the loss immediately after heat stress (*p* > 0.05; Figure 4B,C). For the ST corals, both metrics remained stable at post-heat stress levels (*p* < 0.05) while decline continued over time in the ambient group (*p* < 0.001; Table S7). In *P. verrucosa*, all ambient corals died after heat stress, while 43 and 27% of ST and VT fragments survived, respectively (Figure 4a). All surviving fragments had extensive necrotic areas covered by algae, hampering reliable measurements of photosynthetic efficiency and bleaching. In *A. muricata* and *S. pistillata*, which had the lowest inherent heat stress tolerance (Figure 3, Table 1), all fragments died before the end of the 30 day monitoring period. This included ST and VT fragments, which initially seemed promising, as heat stress responses were milder (Figure 4A).

### Comparative Evaluation of Stress-Hardening Effects of the Thermal Preconditioning Treatments

**Figure 5:**
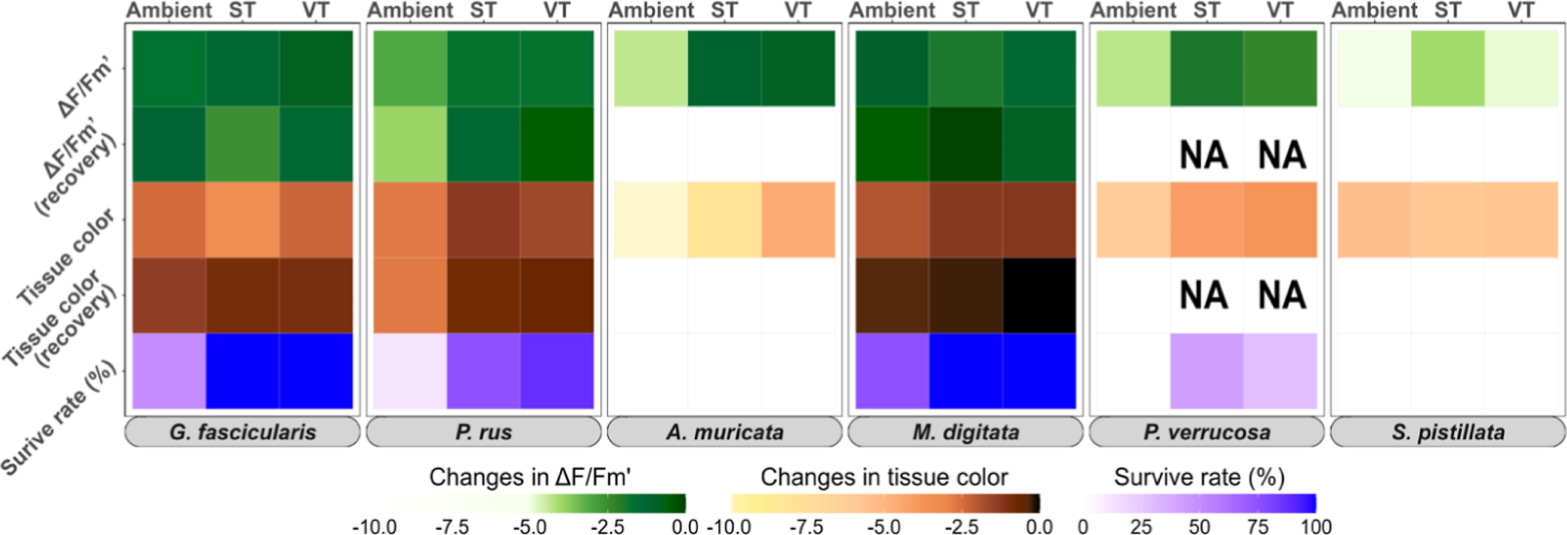
Heat map summarizing stress response physiology, survival, and recovery of six coral species following heat stress. The intensity of change in the measured metrics is coded as the intensity of the colors, with lighter shades indicating a more severe response following heat and darker shades indicating the stability of the metric. Effect sizes of the heat stress responses (Δ*F*/*F*_m_′, tissue color) were determined using the Hedges’ *g* metric as the mean difference between the measurements of the heat and control treatment groups. Values indicate the relative decrease in photosynthetic efficiency (Δ*F*/*F*_m_′, green shades) and bleaching score (tissue color, brown shades) after heat stress and at the end of the recovery period. Kaplan–Meier probabilities represent survival rates (blue to violet shades).

In summary, the ST and VT preconditioning treatments had a stress-hardening effect on corals, increasing the thermal tolerance, survival, and recovery rate of most coral species (Figure 5). Overall, the receptiveness to ST and VT regimes was statistically different only in *A. muricata*, as VT corals bleached less than ST corals (*p* < 0.05). Nonetheless, corals preconditioned with the VT treatment exhibited a slightly higher increase in thermal tolerance (∼10%) and faster recovery than the ST preconditioned corals (Figure 5; Tables S3–S6). This pattern was consistent across most coral species and metrics. The differences between the effects of the ST and VT treatments were smaller than the difference between these two treatments in comparison to the Ambient treatment, where the strongest declines occurred (Figure 5).

## Discussion

We demonstrated that preconditioning with stable-high (ST) and variable-high thermal (VT) regimes successfully stress-hardened corals, enhancing their heat tolerance across species and, in most cases, improving long-term recovery compared to untreated corals in the stable-ambient (Ambient) regime. However, receptiveness to stress-hardening varied across coral species and thermal preconditioning led to a shift in baseline physiology, slightly reducing performance in all branching coral species. These findings highlight the need to consider species-specific traits and potential baseline shifts when designing thermal preconditioning protocols for coral stress hardening.

### Thermal Preconditioning Shifts the Physiological Baselines of Corals

Our data revealed that the branching coral species shifted their physiological baseline in response to thermal preconditioning regimes. These corals were also paler than those from the Ambient control group, indicating a decrease in symbiont cell density and chlorophyll content after the preconditioning phase.^55^ Adjustment of symbiont cell densities is a long-known acclimatization mechanism of corals along temporal and spatial gradients of temperature and light.^65−67^ It is also involved in the modulation of heat stress tolerance, where corals with lower symbiont densities are less susceptible to heat stress than those with high symbiont densities.^52,68,69^ As symbiont numbers decrease, so does the production of hazardous molecules, such as reactive oxygen species during heat stress by these symbionts, linking the initially lower symbiont densities to higher resistance and resilience to heat stress.^52,68,69^

The physiological baseline of massive-growing corals was less sensitive to thermal preconditioning. A naturally low physiological plasticity of these massive corals^70^ could explain their low sensitivity to preconditioning treatments. However, massive-growing coral species have previously also been shown to modulate symbiont densities in response to changes in temperature and light.^71,72^ Additionally, our observation could be explained by their high natural heat stress tolerance,^51^ allowing them to tolerate the specific ST and VT preconditioning treatments without the need to modify their physiological functioning. However, as the differences between growth forms were unexpected, further research with a higher number of species may statistically corroborate these observations and help to uncover the underlying mechanism.

The physiological baseline shift in response to the preconditioning treatments in the branching coral species may provide physiological priming to better cope with heat stress, which may include metabolic changes,^51,73,74^ symbiont flexibility,^17,53^ and enhanced stress-response mechanisms.^14,19^ However, it is also likely to entail trade-offs that affect physiological functioning and productivity in the long term. For instance, the observed reductions in symbiont density and photosynthetic efficiency could be accompanied by reduced skeletal growth rate due to energy resource allocation to tissue growth.^15,52^ Consequently, the energy demands of the holobiont may not be fully covered in the long term.^9,15,74,75^ Therefore, the increase in thermal tolerance should be considered a complex trait that will depend on the amount of energy reserves and the energy budgeting strategy of the corals before and during heat stress phases.^76,77^

Exposure to warmer temperatures may induce shifts in the *Symbiodiniaceae* community toward assemblages dominated by thermo-tolerant species (e.g., *Durusdinium trenchii*).^50,53,78^ In general, such tolerant symbionts are often characterized by a lower photosynthetic activity at ambient temperature compared to the less heat-tolerant symbionts.^50,53,79,80^ They also translocate less carbon to the host and support lower growth rates.^81^ While symbiont communities were not monitored, community shifts are deemed unlikely to occur in all studied distantly related coral species within the short time frame of thermal preconditioning applied in this experiment.^82,83^

### Thermal Preconditioning Enhances Coral Heat-Stress Tolerance

**Figure 6:**
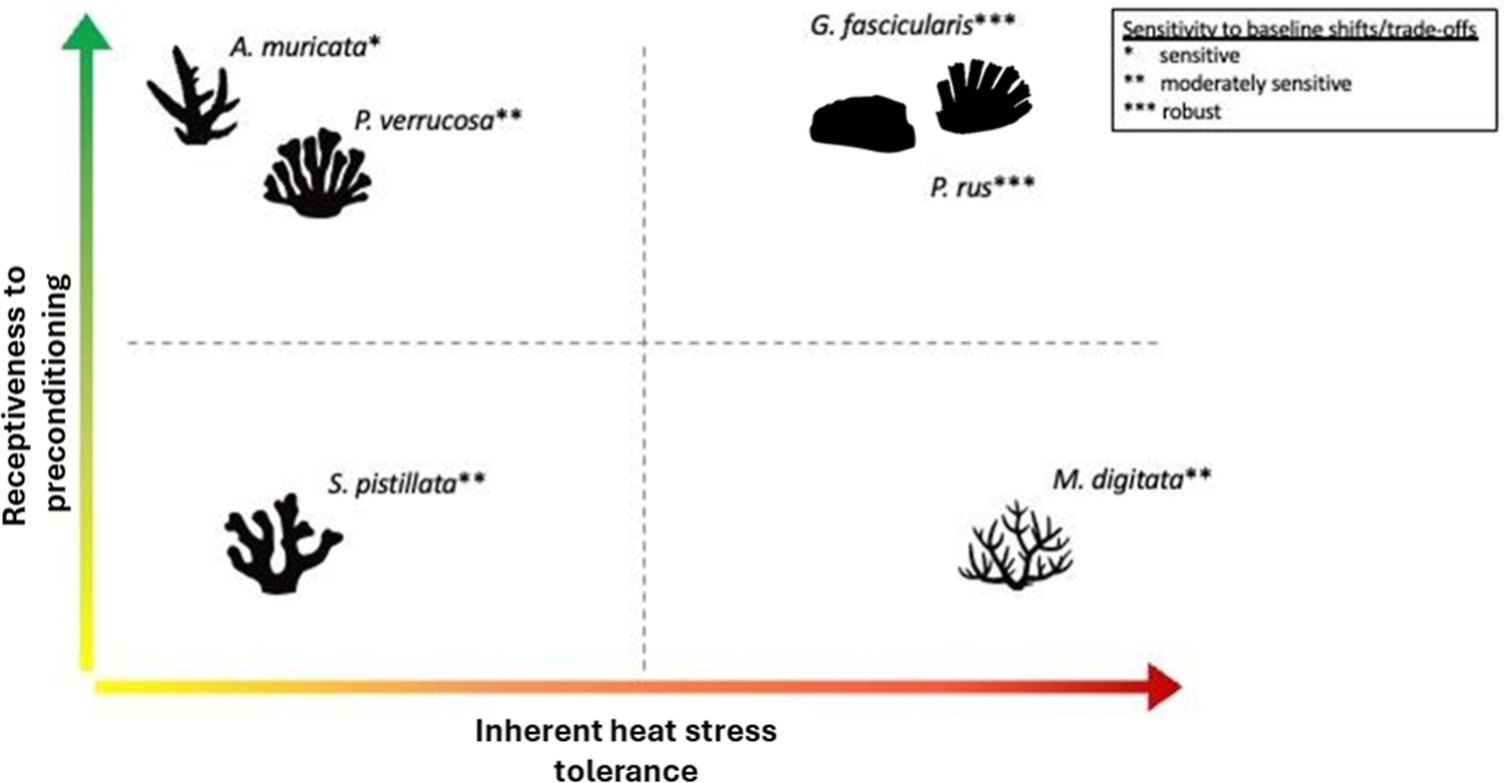
Conceptual representation of the receptiveness to thermal stress-hardening vs the inherent thermal tolerance and susceptibility to baseline shifts through preconditioning in six coral species. The visual representation of each species’ receptiveness to stress-hardening relative to their inherent thermal tolerance provides valuable insights for customizing stress-hardening protocols. Species with high values in both metrics are ideal candidates for restoration programs using stress-hardening, as they can endure extreme thermal anomalies and improve their tolerance to higher temperatures through preconditioning. Species with high receptiveness but low inherent thermal tolerance are also promising, though local environmental conditions, particularly in heat-wave-prone areas, must be considered. Species with high thermal tolerance but low receptiveness to priming may be suitable for restoration programs without thermal priming, as such stress-hardening treatments do not lead to further gains in thermal tolerance. Conversely, species with low tolerance and low receptiveness may not be ideal candidates for such restoration programs. For these species, alternative stress-hardening approaches need to be considered.

Our aquarium experiments demonstrated that thermal preconditioning can be applied to stress-harden corals and enhance their ability to cope with acute heat stress, contributing to the growing body of literature that has reported this phenomenon primarily from the field and including a few ex situ studies.^14,16,18,19,28,34,35^ In some species, thermal tolerance increased by over 80% and massive-growing species were generally more receptive to thermal preconditioning treatments than branching species. Yet, the receptiveness was not related to the inherent thermal tolerance of each coral species. For example, inherently tolerant *M. digitata* was less receptive to preconditioning than other inherently tolerant (*G. fascicularis*) or sensitive species, such as *A. muricata* and *P. verrucosa* (Figure 6). Such interspecific differences in receptiveness to stress-hardening treatments may explain the partially conflicting results found in other studies, especially considering that such investigations were conducted on different coral species.^25,36,37,84^

The specific features of thermal priming conditions, such as the mean exposure temperature and the diel temperature amplitude, are crucial in determining the receptiveness of corals to stress-hardening though preconditioning and increases in stress tolerance. We found that the two preconditioning regimes had similar effects within each coral species. Nonetheless, corals exposed to the variable thermal regime exhibited a greater increase (i.e., approximately 10%) in thermal tolerance. This pattern was consistent across species and response variables, suggesting that thermal variability is a crucial feature to effectively stress-harden corals. This aligns with a growing body of literature documenting that exposure to fluctuating temperature regimes can enhance thermal tolerance.^84^

The individual acclimatization capacity and/or life-history strategy of the coral species must also be taken into account.^70,85−88^ Species with competitive life-history strategies, such as *P. verrucosa* may be more sensitive to extreme temperatures and unable to increase their thermal tolerance as they live near their upper thermal limits. Consequently, preconditioning may be less effective than in weedy or stress-tolerant species such as *P. rus* and *G. fascicularis*. In addition, the stress-tolerant *P. lobata* living in more stable environments exhibited higher thermal tolerance than conspecifics living in environments with higher variability, whereas the competitive species *A. aspera* and the weedy species *P. damicornis* showed the opposite pattern.^34^ Additionally, our results reveal that coral receptiveness to stress-hardening may vary across genotypes. For example, in *G. fascicularis*, all genotypes were receptive to preconditioning. In contrast, other species (e.g., *P. rus*, *P. verrucosa*, and *S. pistillata*) showed a heterogeneous response at the colony level. Notably, inherently more receptive genotypes are a reservoir of adaptive capacity to environmental stressors.^28,89^ Therefore, as receptiveness to thermal preconditioning will largely depend on the type of priming stimulus and the unique thermal tolerance range and life-history traits of each coral species, an empirical assessment of species-specific stress-hardening receptiveness is recommended prior to devising large-scale preconditioning efforts.

### Thermal Preconditioning Enhances Resilience

Our study demonstrated that stress-hardened corals had higher survival and recovery rates 30 days after acute heat stress. Interestingly, the positive effects of the VT treatment excelled over those of the ST treatment. Similarly, other laboratory-based experiments and in situ studies have shown that resilience was superior in corals from high-variability environments compared to other habitats, suggesting that fluctuating temperatures promote metabolic flexibility.^16,21,31,90^

The immediate stress mitigation did not consistently correlate with long-term resilience. Instead, we showed that the increase in resilience was directly linked to the inherent thermal tolerance of each coral species. One explanation for the lower resilience in the thermally sensitive species may be that the heat stress assay exceeded their upper thermal limit, beyond which preconditioning treatments could not further rescue the corals. This aligns with observations in the reef where species from the *Acroporidae* and *Pocilloporidae* families are known to suffer high mortalities during bleaching events and have a weak capacity to recover.^91,92^ Accordingly, in our experiment, the low recovery and high mortality of *A. muricata*, *P. verrucosa*, and *S. pistillata*, might be linked to their lower upper thermal limits.^70,93^ Nevertheless, *Acropora*-dominated intertidal reefs also bleached less and recovered faster than communities from more thermally stable subtidal habitats.^26,90,94^ Reconciling these observations, we conclude that preconditioning treatments, when applied in appropriate doses, have the potential to enhance the resilience of sensitive coral species and can help maintain the stability of their communities. To validate the results from our aquarium experiment, future research should test the effects of preconditioning on coral species in the reef.

During the recovery phase, the higher symbiont performance of ST and VT groups was linked to a significant increase in the survival and recovery rates, suggesting that a limited number of well-performing symbionts may be enough to support coral recovery.^2,95^ Our findings further confirm the work by Middlebrook et al.,^95^ who showed that corals exposed to a priming stimulus exhibited enhanced photosynthetic efficiency during heat stress, while the susceptibility to bleaching remained unchanged. This finding suggests that in stress-hardened corals, symbiont performance may be more important than symbiont density in determining coral resilience.^2,35,95^

### Identifying the Optimal Thermal Regime to Efficiently Stress-Harden Corals

Characteristics of the thermal regime, such as temperature increase, fluctuation amplitude, temperature extremes, and exposure duration are crucial to the success of stress-hardening approaches.^20,28,34,35,96^ In our study, both preconditioning regimes increased the thermal tolerance of corals through exposure to a mean temperature of 29 °C, which is 3 °C above the stable long-term temperature maintained in our aquarium facility. Our results are therefore consistent with previous studies that documented stress-hardening effects at 29 °C (3 °C above the average presummer temperature at the study site in Ka̅ne’ohe Bay),^24^ while an increase of 2 °C was insufficient to trigger such effects.^36^ However, despite the same mean temperatures in our thermal regimes, the diel fluctuations of 3 °C, below the long-term bleaching temperature threshold of 31 °C in the VT treatment,^56^ further increased the thermal tolerance of corals by approximately 10% compared to those in the ST treatment. This aligns with the large body of literature demonstrating that organisms from variable habitats exhibit greater stress tolerance than those from stable habitats^19,21,44,90^ and that the effect is maximized when corals experience a diel thermal variability of 2–3 °C.^34^ This is probably related to enhanced stress mitigation and detoxification mechanisms, as seen in freshwater organisms exposed to heavy metals,^97^ with both temperature maxima and minima influencing priming outcomes.^16,39,44,97^ However, the similar outcomes of the ST and VT preconditioning treatments in our study suggest that the 3 °C temperature increase is the primary driver of the stress-hardening effect. Yet, implementing thermal variability rather than a stable high-temperature regime promises to optimize the stress-hardening effect.

## Data Availability

Data, analyses and visualization are presented in the Supporting Information. All original data and analysis code are accessible via the *GitHub* repository under the accession link: https://github.com/ErikFerrara/Stress-hardening.git.

## Supporting Information Available

Additional information on the pilot study; coral species, countries of origin, and number of colonies used for each species (Table S1); statistical results of thermal preconditioning regime on baseline coral physiology (Table S2); change in effective quantum yield (Δ*F*/*F*_m_′) following heat stress across different preconditioning treatments (Table S3); change in tissue color intensity following heat stress across different preconditioning treatments (Table S4); statistical results of preconditioned corals response to heat stress (Table S5); statistical comparison of coral receptiveness between preconditioning treatments (Table S6); statistical results of the recovery of preconditioned corals after 30 days (Table S7); tissue color changes after preconditioning (Figure S1); tissue color changes after the heat stress assays (Figure S2); corals physiological parameters in the control treatment (Figure S3) (PDF)

## References

[ref1] Coles S. L.; Jokiel P. L. Effects of Temperature on Photosynthesis and Respiration in Hermatypic Corals. Mar. Biol. 1977, 43 (3), 209–216. 10.1007/BF00402313.

[ref2] Fitt W.; Brown B.; Warner M.; Dunne R. Coral Bleaching: Interpretation of Thermal Tolerance Limits and Thermal Thresholds in Tropical Corals. Coral Reefs 2001, 20 (1), 51–65. 10.1007/s003380100146.

[ref3] Frölicher T. L.; Fischer E. M.; Gruber N. Marine Heatwaves under Global Warming. Nature 2018, 560 (7718), 360–364. 10.1038/s41586-018-0383-9. 30111788

[ref4] Oliver E. C. J.; Donat M. G.; Burrows M. T.; Moore P. J.; Smale D. A.; Alexander L. V.; Benthuysen J. A.; Feng M.; Sen Gupta A.; Hobday A. J.; Holbrook N. J.; Perkins-Kirkpatrick S. E.; Scannell H. A.; Straub S. C.; Wernberg T. Longer and More Frequent Marine Heatwaves over the Past Century. Nat. Commun. 2018, 9 (1), 1324 10.1038/s41467-018-03732-9. 29636482 PMC5893591

[ref5] Hughes T. P.; Anderson K. D.; Connolly S. R.; Heron S. F.; Kerry J. T.; Lough J. M.; Baird A. H.; Baum J. K.; Berumen M. L.; Bridge T. C.; Claar D. C.; Eakin C. M.; Gilmour J. P.; Graham N. A. J.; Harrison H.; Hobbs J. P. A.; Hoey A. S.; Hoogenboom M.; Lowe R. J.; McCulloch M. T.; Pandolfi J. M.; Pratchett M.; Schoepf V.; Torda G.; Wilson S. K. Spatial and Temporal Patterns of Mass Bleaching of Corals in the Anthropocene. Science 2018, 359 (6371), 80–83. 10.1126/science.aan8048. 29302011

[ref6] Helgoe J.; Davy S. K.; Weis V. M.; Rodriguez-Lanetty M. Triggers, Cascades, and Endpoints: Connecting the Dots of Coral Bleaching Mechanisms. Biol. Rev. 2024, 99 (3), 715–752. 10.1111/brv.13042. 38217089

[ref7] Coles S. L.; Brown E. Coral Bleaching - Capacity for Acclimatization and Adaptation. Adv. Mar. Biol. 2003, 46, 183–223. 10.1016/S0065-2881(03)46004-5. 14601413

[ref8] Fine M.; Loya Y. Endolithic Algae: An Alternative Source of Photoassimilates during Coral Bleaching. Proc. R. Soc. London, Ser. B 2002, 269 (1497), 1205–1210. 10.1098/rspb.2002.1983. PMC1691023 12065035

[ref9] Wiedenmann J.; D’Angelo C.; Mardones M. L.; Moore S.; Benkwitt C. E.; Graham N. A. J.; Hambach B.; Wilson P. A.; Vanstone J.; Eyal G.; Ben-Zvi O.; Loya Y.; Genin A. Reef-Building Corals Farm and Feed on Their Photosynthetic Symbionts. Nature 2023, 620 (7976), 1018–1024. 10.1038/s41586-023-06442-5. 37612503 PMC10468396

[ref10] Ellegren H.; Sheldon B. C. Genetic Basis of Fitness Differences in Natural Populations. Nature 2008, 452 (7184), 169–175. 10.1038/nature06737. 18337813

[ref11] Voolstra C. R.; Ziegler M. Adapting with Microbial Help: Microbiome Flexibility Facilitates Rapid Responses to Environmental Change. BioEssays 2020, 42 (7), 2000004 10.1002/bies.202000004. 32548850

[ref12] Hilker M.; Schwachtje J.; Baier M.; Balazadeh S.; Bäurle I.; Geiselhardt S.; Hincha D. K.; Kunze R.; Mueller-Roeber B.; Rillig M. C.; Rolff J.; Romeis T.; Schmülling T.; Steppuhn A.; van Dongen J.; Whitcomb S. J.; Wurst S.; Zuther E.; Kopka J. Priming and Memory of Stress Responses in Organisms Lacking a Nervous System. Biol. Rev. 2016, 91 (4), 1118–1133. 10.1111/brv.12215. 26289992

[ref13] DeMerlis A.; Kirkland A.; Kaufman M. L.; Mayfield A. B.; Formel N.; Kolodziej G.; Manzello D. P.; Lirman D.; Traylor-Knowles N.; Enochs I. C. Pre-Exposure to a Variable Temperature Treatment Improves the Response of Acropora Cervicornis to Acute Thermal Stress. Coral Reefs 2022, 41 (2), 435–445. 10.1007/s00338-022-02232-z.

[ref14] Majerova E.; Carey F. C.; Drury C.; Gates R. D. Preconditioning Improves Bleaching Tolerance in the Reef-building Coral Pocillopora Acuta through Modulations in the Programmed Cell Death Pathways. Mol. Ecol. 2021, 30 (14), 3560–3574. 10.1111/mec.15988. 34008873

[ref15] Roik A.; Wall M.; Dobelmann M.; Nietzer S.; Fiesinger A.; Reverter M.; Brefeld D.; Schupp P. J.; Jackson M.; Rutsch M.; Strahl J. Trade-off in a Reef-Building Coral after Six Years of Thermal Acclimation. Sci. Total Environ. 2024, 949, 174589 10.1016/j.scitotenv.2024.174589. 38981551

[ref16] Wall M.; Doering T.; Pohl N.; Putchim L.; Ratanawongwan T.; Roik A. Natural Thermal Stress-Hardening of Corals through Cold Temperature Pulses in the Thai Andaman Sea. bioRxiv 2023, 544549 10.1101/2023.06.12.544549.

[ref17] Ziegler M.; Seneca F. O.; Yum L. K.; Palumbi S. R.; Voolstra C. R. Bacterial Community Dynamics Are Linked to Patterns of Coral Heat Tolerance. Nat. Commun. 2017, 8, 1–8. 10.1038/ncomms14213. 28186132 PMC5309854

[ref18] Ainsworth T. D.; Heron S. F.; Ortiz J. C.; Mumby P. J.; Grech A.; Ogawa D.; Eakin C. M.; Leggat W. Climate Change Disables Coral Bleaching Protection on the Great Barrier Reef. Science 2016, 352 (6283), 338–342. 10.1126/science.aac7125. 27081069

[ref19] Bay R. A.; Palumbi S. R. Rapid Acclimation Ability Mediated by Transcriptome Changes in Reef-Building Corals. Genome Biol. Evol. 2015, 7 (6), 1602–1612. 10.1093/gbe/evv085. 25979751 PMC4494073

[ref20] Middlebrook R.; Hoegh-Guldberg O.; Leggat W. The Effect of Thermal History on the Susceptibility of Reef-Building Corals to Thermal Stress. J. Exp. Biol. 2008, 211 (7), 1050–1056. 10.1242/jeb.013284. 18344478

[ref21] Oliver T. A.; Palumbi S. R. Do Fluctuating Temperature Environments Elevate Coral Thermal Tolerance?. Coral Reefs 2011, 30 (2), 429–440. 10.1007/s00338-011-0721-y.

[ref22] Van Oppen M. J. H.; Oliver J. K.; Putnam H. M.; Gates R. D. Building Coral Reef Resilience through Assisted Evolution. Proc. Natl. Acad. Sci. U.S.A. 2015, 112 (8), 2307–2313. 10.1073/pnas.1422301112. 25646461 PMC4345611

[ref23] Barshis D. J.; Ladner J. T.; Oliver T. A.; Seneca F. O.; Traylor-Knowles N.; Palumbi S. R. Genomic Basis for Coral Resilience to Climate Change. Proc. Natl. Acad. Sci. U.S.A. 2013, 110 (4), 1387–1392. 10.1073/pnas.1210224110. 23297204 PMC3557039

[ref24] Majerová E.; Drury C. Thermal Preconditioning in a Reef-Building Coral Alleviates Oxidative Damage through a BI-1-Mediated Antioxidant Response. Front. Mar. Sci. 2022, 9, 971332 10.3389/fmars.2022.971332.

[ref25] Barshis D. J.; Birkeland C.; Toonen R. J.; Gates R. D.; Stillman J. H. High-Frequency Temperature Variability Mirrors Fixed Differences in Thermal Limits of the Massive Coral Porites Lobata. J. Exp. Biol. 2018, 221 (24), jeb188581 10.1242/jeb.188581. 30305375

[ref26] Camp E. F.; Nitschke M. R.; Rodolfo-Metalpa R.; Houlbreque F.; Gardner S. G.; Smith D. J.; Zampighi M.; Suggett D. J. Reef-Building Corals Thrive within Hot-Acidified and Deoxygenated Waters. Sci. Rep. 2017, 7 (1), 2434 10.1038/s41598-017-02383-y. 28550297 PMC5446402

[ref27] Drury C. Resilience in Reef-Building Corals: The Ecological and Evolutionary Importance of the Host Response to Thermal Stress. Mol. Ecol. 2020, 29 (3), 448–465. 10.1111/mec.15337. 31845413

[ref28] Hackerott S.; Martell H. A.; Eirin-Lopez J. M. Coral Environmental Memory: Causes, Mechanisms, and Consequences for Future Reefs. Trends Ecol. Evol. 2021, 36 (11), 1011–1023. 10.1016/j.tree.2021.06.014. 34366170

[ref29] Marhoefer S. R.; Zenger K. R.; Strugnell J. M.; Logan M.; van Oppen M. J. H.; Kenkel C. D.; Bay L. K. Signatures of Adaptation and Acclimatization to Reef Flat and Slope Habitats in the Coral Pocillopora Damicornis. Front. Mar. Sci. 2021, 8, 704709 10.3389/fmars.2021.704709.

[ref30] Marzonie M. R.; Bay L. K.; Bourne D. G.; Hoey A. S.; Matthews S.; Nielsen J. J. V.; Harrison H. B. The Effects of Marine Heatwaves on Acute Heat Tolerance in Corals. Glob. Change Biol. 2023, 29 (2), 404–416. 10.1111/gcb.16473. PMC10092175 36285622

[ref31] Schoepf V.; Stat M.; Falter J. L.; McCulloch M. T. Limits to the Thermal Tolerance of Corals Adapted to a Highly Fluctuating, Naturally Extreme Temperature Environment. Sci. Rep. 2015, 5 (May), 1–14. 10.1038/srep17639. PMC4667274 26627576

[ref32] Schoepf V.; Jung M. U.; McCulloch M. T.; White N. E.; Stat M.; Thomas L. Thermally Variable, Macrotidal Reef Habitats Promote Rapid Recovery From Mass Coral Bleaching. Front. Mar. Sci. 2020, 7, 245 10.3389/fmars.2020.00245.

[ref33] Palumbi S. R.; Barshis D. J.; Traylor-Knowles N.; Bay R. A. Mechanisms of Reef Coral Resistance to Future Climate Change. Science 2014, 344 (6186), 895–898. 10.1126/science.1251336. 24762535

[ref34] Brown K. T.; Martynek M. P.; Barott K. L. Local Habitat Heterogeneity Rivals Regional Differences in Coral Thermal Tolerance. Coral Reefs 2024, 43 (3), 571–585. 10.1007/s00338-024-02484-x.

[ref35] Martell H. A. Thermal Priming and Bleaching Hormesis in the Staghorn Coral, Acropora Cervicornis (Lamarck 1816). J. Exp. Mar. Biol. Ecol. 2023, 560, 151820 10.1016/j.jembe.2022.151820.

[ref36] Henley E. M.; Bouwmeester J.; Jury C. P.; Toonen R. J.; Quinn M.; Lager C. V. A.; Hagedorn M. Growth and Survival among Hawaiian Corals Outplanted from Tanks to an Ocean Nursery Are Driven by Individual Genotype and Species Differences Rather than Preconditioning to Thermal Stress. PeerJ 2022, 10 (e13112), e13112 10.7717/peerj.13112. 35345587 PMC8957268

[ref37] Klepac C. N.; Barshis D. J. Reduced Thermal Tolerance of Massive Coral Species in a Highly Variable Environment: Reduced Heat Tolerance of Massive Corals. Proc. R. Soc. B 2020, 287 (1933), 19–21. 10.1098/rspb.2020.1379. PMC7482266 32811319

[ref38] Putnam H. M.; Edmunds P. J. The Physiological Response of Reef Corals to Diel Fluctuations in Seawater Temperature. J. Exp. Mar. Biol. Ecol. 2011, 396 (2), 216–223. 10.1016/j.jembe.2010.10.026.

[ref39] Schoepf V.; Carrion S. A.; Pfeifer S. M.; Naugle M.; Dugal L.; Bruyn J.; McCulloch M. T. Stress-Resistant Corals May Not Acclimatize to Ocean Warming but Maintain Heat Tolerance under Cooler Temperatures. Nat. Commun. 2019, 10 (1), 4031 10.1038/s41467-019-12065-0. 31530800 PMC6748961

[ref40] Thomas L.; Rose N. H.; Bay R. A.; López E. H.; Morikawa M. K.; Ruiz-Jones L.; Palumbi S. R. Mechanisms of Thermal Tolerance in Reef-Building Corals across a Fine-Grained Environmental Mosaic: Lessons from Ofu, American Samoa. Front. Mar. Sci. 2018, 4, 434 10.3389/fmars.2017.00434.

[ref41] Calabrese E. J.; Bachmann K. A.; Bailer A. J.; Bolger P. M.; Borak J.; Cai L.; Cedergreen N.; Cherian M. G.; Chiueh C. C.; Clarkson T. W.; Cook R. R.; Diamond D. M.; Doolittle D. J.; Dorato M. A.; Duke S. O.; Feinendegen L.; Gardner D. E.; Hart R. W.; Hastings K. L.; Hayes A. W.; Hoffmann G. R.; Ives J. A.; Jaworowski Z.; Johnson T. E.; Jonas W. B.; Kaminski N. E.; Keller J. G.; Klaunig J. E.; Knudsen T. B.; Kozumbo W. J.; Lettieri T.; Liu S.-Z.; Maisseu A.; Maynard K. I.; Masoro E. J.; McClellan R. O.; Mehendale H. M.; Mothersill C.; Newlin D. B.; Nigg H. N.; Oehme F. W.; Phalen R. F.; Philbert M. A.; Rattan S. I. S.; Riviere J. E.; Rodricks J.; Sapolsky R. M.; Scott B. R.; Seymour C.; Sinclair D. A.; Smith-Sonneborn J.; Snow E. T.; Spear L.; Stevenson D. E.; Thomas Y.; Tubiana M.; Williams G. M.; Mattson M. P. Biological Stress Response Terminology: Integrating the Concepts of Adaptive Response and Preconditioning Stress within a Hormetic Dose–Response Framework. Toxicol. Appl. Pharmacol. 2007, 222 (1), 122–128. 10.1016/j.taap.2007.02.015. 17459441

[ref42] Calabrese E. J.; Mattson M. P. How Does Hormesis Impact Biology, Toxicology, and Medicine?. npj Aging Mech. Dis. 2017, 3 (1), 1–8. 10.1038/s41514-017-0013-z. 28944077 PMC5601424

[ref43] Carelli G.; Iavicoli I. Defining Hormesis: The Necessary Tool to Clarify Experimentally the Low Dose–Response Relationship. Hum. Exp. Toxicol. 2002, 21 (2), 103–104. 10.1191/0960327102ht219oa. 12102492

[ref44] Drury C.; Dilworth J.; Majerová E.; Caruso C.; Greer J. B. Expression Plasticity Regulates Intraspecific Variation in the Acclimatization Potential of a Reef-Building Coral. Nat. Commun. 2022, 13 (1), 4790 10.1038/s41467-022-32452-4. 35970904 PMC9378650

[ref45] Bertucci A.; Forêt S.; Ball E. E.; Miller D. J. Transcriptomic Differences between Day and Night in *Acropora Millepora* Provide New Insights into Metabolite Exchange and Light-Enhanced Calcification in Corals. Mol. Ecol. 2015, 24 (17), 4489–4504. 10.1111/mec.13328. 26198296

[ref46] Kenkel C. D.; Almanza A. T.; Matz M. V. Fine-Scale Environmental Specialization of Reef-Building Corals Might Be Limiting Reef Recovery in the Florida Keys. Ecology 2015, 96 (12), 3197–3212. 10.1890/14-2297.1. 26909426

[ref47] Rivest E. B.; Comeau S.; Cornwall C. E. The Role of Natural Variability in Shaping the Response of Coral Reef Organisms to Climate Change. Curr. Clim. Change Rep. 2017, 3 (4), 271–281. 10.1007/s40641-017-0082-x.

[ref48] Roper C. D.; Donelson J. M.; Ferguson S.; van Oppen M. J. H.; Cantin N. E. Long-Term Preconditioning of the Coral Pocillopora Acuta Does Not Restore Performance in Future Ocean Conditions. Coral Reefs 2023, 42 (5), 1079–1096. 10.1007/s00338-023-02401-8.

[ref49] Evensen N. R.; Voolstra C. R.; Fine M.; Perna G.; Buitrago-López C.; Cárdenas A.; Banc-Prandi G.; Rowe K.; Barshis D. J. Empirically Derived Thermal Thresholds of Four Coral Species along the Red Sea Using a Portable and Standardized Experimental Approach. Coral Reefs 2022, 41 (2), 239–252. 10.1007/s00338-022-02233-y.

[ref50] Cunning R.; Silverstein R. N.; Baker A. C. Symbiont Shuffling Linked to Differential Photochemical Dynamics of Symbiodinium in Three Caribbean Reef Corals. Coral Reefs 2018, 37 (1), 145–152. 10.1007/s00338-017-1640-3.

[ref51] Jurriaans S.; Hoogenboom M. O. Seasonal Acclimation of Thermal Performance in Two Species of Reef-Building Corals. Mar. Ecol.: Prog. Ser. 2020, 635, 55–70. 10.3354/meps13203.

[ref52] Cornwell B.; Armstrong K.; Walker N. S.; Lippert M.; Nestor V.; Golbuu Y.; Palumbi S. R. Widespread Variation in Heat Tolerance and Symbiont Load Are Associated with Growth Tradeoffs in the Coral Acropora Hyacinthus in Palau. eLife 2021, 10, e64790 10.7554/eLife.64790. 34387190 PMC8457836

[ref53] Williamson O. M.; Allen C. E.; Williams D. E.; Johnson M. W.; Miller M. W.; Baker A. C. Neighboring Colonies Influence Uptake of Thermotolerant Endosymbionts in Threatened Caribbean Coral Recruits. Coral Reefs 2021, 40 (3), 867–879. 10.1007/s00338-021-02090-1.

[ref54] Voolstra C. R.; Buitrago-López C.; Perna G.; Cárdenas A.; Hume B. C. C.; Rädecker N.; Barshis D. J. Standardized Short-Term Acute Heat Stress Assays Resolve Historical Differences in Coral Thermotolerance across Microhabitat Reef Sites. Glob. Change Biol. 2020, 26 (8), 4328–4343. 10.1111/gcb.15148. 32567206

[ref55] Ferrara E. F.; Bauer L.; Puntin G.; Bautz F. R.; Celayir S.; Do M.-S.; Eck F. L.; Heider M. C.; Wissel P. M.-C.; Arnold A.; Wilke T.; Reichert J.; Ziegler M. RGB Color Indices as Proxy for Symbiont Cell Density and Chlorophyll Content during Coral Bleaching. bioRxiv 2024, 2024.12.20.629333 10.1101/2024.12.20.629333.

[ref56] Reichert J.; Tirpitz V.; Anand R.; Bach K.; Knopp J.; Schubert P.; Wilke T.; Ziegler M. Interactive Effects of Microplastic Pollution and Heat Stress on Reef-Building Corals. Environ. Pollut. 2021, 290, 118010 10.1016/j.envpol.2021.118010. 34488160

[ref57] Doering T.; Wall M.; Putchim L.; Rattanawongwan T.; Schroeder R.; Hentschel U.; Roik A. Towards Enhancing Coral Heat Tolerance: A “Microbiome Transplantation” Treatment Using Inoculations of Homogenized Coral Tissues. Microbiome 2021, 9 (1), 102 10.1186/s40168-021-01053-6. 33957989 PMC8103578

[ref58] Evensen N. R.; Fine M.; Perna G.; Voolstra C. R.; Barshis D. J. Remarkably High and Consistent Tolerance of a Red Sea Coral to Acute and Chronic Thermal Stress Exposures. Limnol. Oceanogr. 2021, 66 (5), 1718–1729. 10.1002/lno.11715.

[ref59] Walker N. S.; Nestor V.; Golbuu Y.; Palumbi S. R. Coral Bleaching Resistance Variation Is Linked to Differential Mortality and Skeletal Growth during Recovery. Evol. Appl. 2023, 16 (2), 504–517. 10.1111/eva.13500. 36793702 PMC9923480

[ref60] Wickham H. Getting Started with Ggplot2. In ggplot2: Elegant Graphics for Data Analysis; Wickham H., Ed.; Springer International Publishing: Cham, 2016; pp 11–31. 10.1007/978-3-319-24277-4_2.

[ref61] Ho J.; Tumkaya T.; Aryal S.; Choi H.; Claridge-Chang A. Moving beyond P Values: Data Analysis with Estimation Graphics. Nat. Methods 2019, 16 (7), 565–566. 10.1038/s41592-019-0470-3. 31217592

[ref62] Bates D.; Mächler M.; Bolker B.; Walker S. Fitting Linear Mixed-Effects Models Using Lme4. J. Stat. Software 2015, 67, 1–48. 10.18637/jss.v067.i01.

[ref63] Fox J.; Weisberg S. R. Companion 3E. https://www.john-fox.ca/Companion/index.html (accessed 10 18, 2023).

[ref64] Therneau T. M.; Lumley T.; Atkinson E.; Crowson C. Package for Survival Analysis in R. Version 3.4 [Computer Software], 2024. (accessed 2023 10 18). 10.32614/CRAN.package.survival.

[ref65] Fagoonee I.; Wilson H. B.; Hassell M. P.; Turner J. R. The Dynamics of Zooxanthellae Populations: A Long-Term Study in the Field. Science 1999, 283 (5403), 843–845. 10.1126/science.283.5403.843. 9933167

[ref66] Fitt W. K.; McFarland F. K.; Warner M. E.; Chilcoat G. C. Seasonal Patterns of Tissue Biomass and Densities of Symbiotic Dinoflagellates in Reef Corals and Relation to Coral Bleaching. Limnol. Oceanogr. 2000, 45 (3), 677–685. 10.4319/lo.2000.45.3.0677.

[ref67] Stimson J. The Annual Cycle of Density of Zooxanthellae in the Tissues of Field and Laboratory-Held *Pocillopora Damicornis* (Linnaeus). J. Exp. Mar. Biol. Ecol. 1997, 214 (1), 35–48. 10.1016/S0022-0981(96)02753-0.

[ref68] Cunning R.; Baker A. C. Excess Algal Symbionts Increase the Susceptibility of Reef Corals to Bleaching. Nat. Clim. Change 2013, 3 (3), 259–262. 10.1038/nclimate1711.

[ref69] Cunning R.; Baker A. C. Not Just Who, but How Many: The Importance of Partner Abundance in Reef Coral Symbioses. Front. Microbiol. 2014, 5, 400 10.3389/fmicb.2014.00400. 25136339 PMC4120693

[ref70] Darling E. S.; Alvarez-Filip L.; Oliver T. A.; McClanahan T. R.; Côté I. M. Evaluating Life-History Strategies of Reef Corals from Species Traits. Ecol. Lett. 2012, 15 (12), 1378–1386. 10.1111/j.1461-0248.2012.01861.x. 22938190

[ref71] Sawall Y.; Nicosia A. M.; McLaughlin K.; Ito M. Physiological Responses and Adjustments of Corals to Strong Seasonal Temperature Variations (20–28°C). J. Exp. Biol. 2022, 225 (13), jeb244196 10.1242/jeb.244196. 35702952

[ref72] Ziegler M.; Roder C.; Büchel C.; Voolstra C. R. Niche Acclimatization in Red Sea Corals Is Dependent on Flexibility of Host-Symbiont Association. Mar. Ecol.: Prog. Ser. 2015, 533, 149–161. 10.3354/meps11365.

[ref73] García F. C.; Osman E. O.; Garcias-Bonet N.; Delgadillo-Ordoñez N.; Santoro E. P.; Raimundo I.; Villela H. D. M.; Voolstra C. R.; Peixoto R. S. Seasonal Changes in Coral Thermal Threshold Suggest Species-Specific Strategies for Coping with Temperature Variations. Commun. Biol. 2024, 7 (1), 1–8. 10.1038/s42003-024-07340-w. 39702455 PMC11659276

[ref74] Gibbin E. M.; Krueger T.; Putnam H. M.; Barott K. L.; Bodin J.; Gates R. D.; Meibom A. Short-Term Thermal Acclimation Modifies the Metabolic Condition of the Coral Holobiont. Front. Mar. Sci. 2018, 5, 1–11. 10.3389/fmars.2018.00010.29552559

[ref75] Rodrigues L. J.; Grottoli A. G. Energy Reserves and Metabolism as Indicators of Coral Recovery from Bleaching. Limnol. Oceanogr. 2007, 52 (5), 1874–1882. 10.4319/lo.2007.52.5.1874.

[ref76] Grottoli A. G.; Rodrigues L. J.; Palardy J. E. Heterotrophic Plasticity and Resilience in Bleached Corals. Nature 2006, 440 (7088), 1186–1189. 10.1038/nature04565. 16641995

[ref77] Huffmyer A. S.; Johnson C. J.; Epps A. M.; Lemus J. D.; Gates R. D. Feeding and Thermal Conditioning Enhance Coral Temperature Tolerance in Juvenile Pocillopora Acuta. R. Soc. Open Sci. 2021, 8 (5), 210644 10.1098/rsos.210644. 34084554 PMC8150050

[ref78] Silverstein R. N.; Cunning R.; Baker A. C. Change in Algal Symbiont Communities after Bleaching, Not Prior Heat Exposure, Increases Heat Tolerance of Reef Corals. Glob. Change Biol. 2015, 21 (1), 236–249. 10.1111/gcb.12706. 25099991

[ref79] Cantin N. E.; van Oppen M. J. H.; Willis B. L.; Mieog J. C.; Negri A. P. Juvenile Corals Can Acquire More Carbon from High-Performance Algal Symbionts. Coral Reefs 2009, 28 (2), 405–414. 10.1007/s00338-009-0478-8.

[ref80] Quigley K. M.; Randall C. J.; van Oppen M. J. H.; Bay L. K. Assessing the Role of Historical Temperature Regime and Algal Symbionts on the Heat Tolerance of Coral Juveniles. Biol. Open 2020, 9 (1), bio047316 10.1242/bio.047316. 31915210 PMC6994947

[ref81] Pettay D. T.; Wham D. C.; Smith R. T.; Iglesias-Prieto R.; LaJeunesse T. C. Microbial Invasion of the Caribbean by an Indo-Pacific Coral Zooxanthella. Proc. Natl. Acad. Sci. U.S.A. 2015, 112 (24), 7513–7518. 10.1073/pnas.1502283112. 26034268 PMC4475936

[ref82] Boulotte N. M.; Dalton S. J.; Carroll A. G.; Harrison P. L.; Putnam H. M.; Peplow L. M.; van Oppen M. J. H. Exploring the Symbiodinium Rare Biosphere Provides Evidence for Symbiont Switching in Reef-Building Corals. ISME J. 2016, 10 (11), 2693–2701. 10.1038/ismej.2016.54. 27093048 PMC5113844

[ref83] Scharfenstein H. J.; Chan W. Y.; Buerger P.; Humphrey C.; van Oppen M. J. H. Evidence for de Novo Acquisition of Microalgal Symbionts by Bleached Adult Corals. ISME J. 2022, 16 (6), 1676–1679. 10.1038/s41396-022-01203-0. 35132118 PMC9122906

[ref84] Schoepf V.; Sanderson H.; Larcombe E. Coral Heat Tolerance under Variable Temperatures: Effects of Different Variability Regimes and Past Environmental History vs. Current Exposure. Limnol. Oceanogr. 2022, 67 (2), 404–418. 10.1002/lno.12000.

[ref85] Brown K. T.; Eyal G.; Dove S. G.; Barott K. L. Fine-Scale Heterogeneity Reveals Disproportionate Thermal Stress and Coral Mortality in Thermally Variable Reef Habitats during a Marine Heatwave. Coral Reefs 2023, 42 (1), 131–142. 10.1007/s00338-022-02328-6. 36415309 PMC9672654

[ref86] Dilworth J.; Caruso C.; Kahkejian V. A.; Baker A. C.; Drury C. Host Genotype and Stable Differences in Algal Symbiont Communities Explain Patterns of Thermal Stress Response of Montipora Capitata Following Thermal Pre-Exposure and across Multiple Bleaching Events. Coral Reefs 2021, 40 (1), 151–163. 10.1007/s00338-020-02024-3.

[ref87] Kenkel C. D.; Goodbody-Gringley G.; Caillaud D.; Davies S. W.; Bartels E.; Matz M. V. Evidence for a Host Role in Thermotolerance Divergence between Populations of the Mustard Hill Coral (Porites Astreoides) from Different Reef Environments. Mol. Ecol. 2013, 22 (16), 4335–4348. 10.1111/mec.12391. 23906315

[ref88] Kenkel C. D.; Matz M. V. Gene Expression Plasticity as a Mechanism of Coral Adaptation to a Variable Environment. Nat. Ecol. Evol. 2017, 1 (1), 1–6. 10.1038/s41559-016-0014. 28812568

[ref89] Million W. C.; Ruggeri M.; O’Donnell S.; Bartels E.; Conn T.; Krediet C. J.; Kenkel C. D. Evidence for Adaptive Morphological Plasticity in the Caribbean Coral, Acropora Cervicornis. Proc. Natl. Acad. Sci. U.S.A. 2022, 119 (49), e2203925119 10.1073/pnas.2203925119. 36442118 PMC9894258

[ref90] Padilla-Gamiño J. L.; Timmins-Schiffman E.; Lenz E. A.; White S. J.; Axworthy J.; Potter A.; Lopez J.; Wang F. Coral Long-Term Recovery after Bleaching: Implications for Sexual Reproduction and Physiology. bioRxiv 2024, 2024.04.09.588789 10.1101/2024.04.09.588789.

[ref91] Baird A. H.; Marshall P. A. Mortality, Growth and Reproduction in Scleractinian Corals Following Bleaching on the Great Barrier Reef. Mar. Ecol.: Prog. Ser. 2002, 237, 133–141. 10.3354/meps237133.

[ref92] Burt J.; Al-Harthi S.; Al-Cibahy A. Long-Term Impacts of Coral Bleaching Events on the World’s Warmest Reefs. Mar. Environ. Res. 2011, 72 (4), 225–229. 10.1016/j.marenvres.2011.08.005. 21880360

[ref93] McClanahan T. R.; Ateweberhan M.; Graham N. a. J.; Wilson S. K.; Sebastián C. R.; Guillaume M. M. M.; Bruggemann J. H. Western Indian Ocean Coral Communities: Bleaching Responses and Susceptibility to Extinction. Mar. Ecol.: Prog. Ser. 2007, 337, 1–13. 10.3354/meps337001.

[ref94] Le Nohaïc M.; Ross C. L.; Cornwall C. E.; Comeau S.; Lowe R.; McCulloch M. T.; Schoepf V. Marine Heatwave Causes Unprecedented Regional Mass Bleaching of Thermally Resistant Corals in Northwestern Australia. Sci. Rep. 2017, 7 (1), 14999 10.1038/s41598-017-14794-y. 29101362 PMC5670227

[ref95] Middlebrook R.; Anthony K. R. N.; Hoegh-Guldberg O.; Dove S. Thermal Priming Affects Symbiont Photosynthesis but Does Not Alter Bleaching Susceptibility in Acropora Millepora. J. Exp. Mar. Biol. Ecol. 2012, 432–433, 64–72. 10.1016/j.jembe.2012.07.005.

[ref96] Brown K.; Barott K. L. The Costs and Benefits of Environmental Memory for Reef-Building Corals Coping with Recurring Marine Heatwaves. Integr. Comp. Biol. 2022, 62 (6), 1748–1755. 10.1093/icb/icac074. 35661887

[ref97] Hallman T. A.; Brooks M. L. The Deal with Diel: Temperature Fluctuations, Asymmetrical Warming, and Ubiquitous Metals Contaminants. Environ. Pollut. 2015, 206, 88–94. 10.1016/j.envpol.2015.06.005. 26142755

