# Supplementary material for: Ex Situ Thermal Preconditioning Modulates Coral Physiology and Enhances Heat Tolerance: A Multispecies Perspective for Active Restoration
Source: Environ Sci Technol. 2025 Apr 25;59(17):8527–40. doi: 10.1021/acs.est.4c08640 (PMC12060272; doi:10.1021/acs.est.4c08640)
Supplement: Supplementary file 1 — es4c08640_si_001.pdf [file es4c08640_si_001.pdf]

Supporting information for

Ex-situ thermal preconditioning modulates coral physiology and enhances heat tolerance: A multi-species perspective for active restoration

Erik F. Ferrara[1], Anna Roik[2,3], Franziska Wöhrmann-Zipf[1], Maren Ziegler[1*]

[1]Marine Holobiomics Lab, Justus Liebig University Giessen, Department of Animal Ecology and Systematics, Heinrich-Buff-Ring 26-32 IFZ, 35392 Giessen, Germany
[2]Helmholtz Institute for Functional Marine Biodiversity at the University of Oldenburg (HIFMB), Oldenburg, Germany
[3]Alfred Wegener Institute, Helmholtz Center for Polar and Marine Research, Bremerhaven, Germany

Corresponding author: maren.ziegler@bio.uni-giessen.de

Number of pages: 12
Number of tables: 7
Number of figures: 3

List of supplementary contents

Supporting Information on the pilot study S. 2
Table S1: Coral species, countries of origin, and number of colonies used for each species. **S. 3**
Table S2: Statistical results of thermal preconditioning regime on baseline coral physiology. **S. 4**
Table S3: Change in effective quantum yield (ΔF/Fm′) following heat stress across different preconditioning treatments. **S. 5**
Table S4: Change in tissue color intensity following heat stress across different preconditioning treatments. **S. 6**
Table S5: Statistical results of preconditioned corals response to heat stress. **S. 7**
Table S6: Statistical comparison of coral receptiveness between preconditioning treatments. **S. 8**
Table S7: Statistical results of the recovery of preconditioned corals after 30 days. **S. 9**
Figure S1: Tissue color changes after preconditioning. **S. 10**
Figure S2: Tissue color changes after the heat stress assays. **S. 11**
Figure S3: Corals physiological parameters in the Control treatment. **S. 12**

Supporting Information on the pilot study

The target temperatures for the heat stress assays to induce heat stress in the different coral species were determined in a pilot heat stress series. This pilot study aimed to identify the specific temperatures that would cause a severe but not lethal response in each of the coral species. This ideal heat stress temperature can differ between species of different heat tolerance levels. To this end, test fragments from each species were exposed to 34 °C, 35 °C, or 36 °C following two established protocols (Doering et al., 2021; Reichert et al., 2021). We observed a measurable increase in the heat stress responses between 34° and 35°C, while 36°C was lethal for most species. Based on the observations in this pilot study, we selected the target temperature of 35 °C, i.e., 9 °C above the ambient mean temperature of the facility.

Supplementary Tables

Table S1: Coral species, countries of origin, and number of colonies used for each species during the experiment

Families	Species	Colony origin	N. of colonies
Acroporidae	*Acropora muricata*	Indonesia	4
Acroporidae	*Acropora muricata*	Australia	4
Acroporidae	*Montipora digitata*	Indonesia	4
Euphylliidae	*Galaxea fascicularis*	Red Sea	4
Pocilloporidae	*Pocillopora verrucosa*	Red Sea	4
Pocilloporidae	*Pocillopora verrucosa*	Indonesia	3
Pocilloporidae	*Stylophora pistillata*	Fiji	5
Poritidae	*Porites rus*	Red Sea	4
Poritidae	*Porites rus*	Indo-Pacific	4

Table S2: Statistical results of thermal preconditioning regime on baseline coral physiology. The F statistic was calculated using the ANOVA test on the linear mixed-effects model. Effect size, shown as hedges g, was estimated as the difference between treatments comparing the raw values of the Ambient group against the two preconditioning treatments.

**Species**	**Trait**	***Term***	***F***	***p***	**Post-hoc comparison**	**Effect Size (Hedges's g)**	***P* values**
G. fascicularis	ΔF/Fm'	Preconditioning	0.13	0.879			
	Tissue color	Preconditioning	0.761	0.474			
P. rus	ΔF/Fm'	Preconditioning	5.275	**0.007**	ST - Ambient	0.22	n.s.
					VT - Ambient	0.61	**< 0.01**
	Tissue color	Preconditioning	3.023	0.0539			
A. muricata	ΔF/Fm'	Preconditioning	22.458	**<0.001**	ST - Ambient	-2.55	**< 0.001**
					VT - Ambient	-2.68	**< 0.001**
					ST - VT	NA	**< 0.01**
	Tissue color	Preconditioning	45.902	**<0.001**	ST - Ambient	-0.63	**< 0.05**
					VT -Ambient	-1.99	**<0.001**
					ST - VT	NA	**<0.001**
M. digitata	ΔF/Fm'	Preconditioning	2.19	0.1246			
	Tissue color	Preconditioning	17.896	**<0.001**	ST - Ambient	-1.09	**<0.001**
					VT -Ambient	-0.62	**<0.001**
P. verrucosa	ΔF/Fm'	Preconditioning	15.693	**<0.001**	ST - Ambient	-1.04	**<0.001**
					VT -Ambient	-0.98	**<0.001**
	Tissue color	Preconditioning	12.006	**< 0.001**	ST - Ambient	-0.81	**0.006**
					VT -Ambient	-1,00	**<0.001**
S. pistillata	ΔF/Fm'	Preconditioning	7.327	**0.0015**	ST - Ambient	-1.22	**<0.001**
					VT -Ambient	-0.79	**<0.05**
	Tissue color	Preconditioning	7.922	**0.0009**	ST - Ambient	-0.60	**<0.001**
					VT -Ambient	-0.22	0.304
					ST - VT	NA	**<0.05**

Table S3: Change in effective quantum yield (ΔF/Fm′) following heat stress across different preconditioning treatments. For each coral species, ΔAmbient, ΔST, and ΔVT represent the mean change in effective quantum yield (paired post-heat value minus post-preconditioning) in the Ambient, ST, and VT groups, respectively. Percentage values indicate heat stress mitigation, expressed as the relative reduction ((Δambient – ΔST or ΔVT)/ |Δambient|) of ΔF/Fm′ in the ST and VT treatments compared to the Ambient group (i.g, in *G. fascicularis*, the decline in effective quantum yield was approximately 90% less pronounced in ST corals compared to the Ambient group). The final column shows the percentage difference in stress response between VT and ST treatments. The most effective preconditioning treatments for each species are highlighted in bold.

Species	ΔAmbient	ΔST	ΔVT	ΔST minus ΔAmbient (%)	ΔVT minus ΔAmbient (%)	VT minus ST (%)
G. fascicularis	-0.128	-0.012	-0.015	**-90.66**	-88.72	-1.95
P. rus	-0.459	-0.255	-0.195	-44.34	**-57.41**	**+13.07**
A. muricata	-0.378	-0.078	-0.069	-79.52	**-81.64**	**+2.11**
M. digitata	-0.071	-0.047	-0.028	-33.57	**-60.14**	**+26.57**
P. verrucosa	-0.458	-0.253	-0.188	-44.76	**-58.84**	**+14.08**
S. pistillata	-0.551	-0.408	-0.382	-26.04	**-30.67**	**+4.63**

Table S4: Change in tissue color intensity following heat stress across different preconditioning treatments. For each coral species, ΔAmbient, ΔST, and ΔVT represent the mean change in effective quantum yield (paired post-heat value minus post-preconditioning) in the Ambient, ST, and VT groups, respectively. Percentage values indicate heat stress mitigation, expressed as the relative reduction ((Δambient – ΔST or ΔVT)/ |Δambient|) of tissue color in the ST and VT treatments compared to the Ambient group. The final column shows the percentage difference in stress response between VT and ST treatments. The most effective preconditioning treatments for each species are highlighted in bold.

Species	ΔAmbient	ΔST	ΔVT	ΔST minus ΔAmbient (%)	ΔVT minus ΔAmbient (%)	VT minus ST (%)
G. fascicularis	-60.973	-28.178	-26.610	-53.79	**-56.36**	**+2.57**
P. rus	-38.454	-27.802	-31.386	**-27.70**	-18.38	-9.32
A. muricata	-128.826	-98.456	-86.824	-23.58	**-32.60**	**+9.03**
M. digitata	-43.772	-22.870	-25.168	**-47.75**	-42.50	-5.25
P. verrucosa	-87.950	-69.782	-61.224	-20.66	**-30.39**	**+9.73**
S. pistillata	-103.238	-96.879	-98.204	**-6.16**	-4.88	-1.28

Table S5: Statistical results of preconditioned corals response to heat stress (post-preconditioning vs. heat). Significant *p*-values (Wilcoxon tests) are shown in bold. Effect size, shown as Hedges g, was estimated within each treatment by comparing post-preconditioning against post-heat paired values.

Species	Analysis	Term	Contrast (post-heat - post-preconditioning)	*P value (Wilcoxon test)*	Signif.	Effect size
G. fascicularis	ΔF/Fm'	Preconditioning	Ambient	**<0.001**	***	-1.70
			ST	**0.01**	**	-1.35
			VT	0.103		-0.85
	Tissue color	Preconditioning	Ambient	**<0.001**	***	-2.43
			ST	**<0.001**	***	-3.42
			VT	**0.001**	***	-2.32
P. rus	ΔF/Fm'	Preconditioning	Ambient	**<0.001**	***	-3.08
			ST	**<0.001**	***	-1.77
			VT	**<0.001**	***	-1.76
	Tissue color	Preconditioning	Ambient	**<0.001**	***	-2.72
			ST	**<0.001**	***	-1.28
			VT	**<0.001**	***	-1.65
A. muricata	ΔF/Fm'	Preconditioning	Ambient	**<0.001**	***	-4.35
			ST	**<0.01**	**	-1.16
			VT	**<0.05**	*	-0.9
	Tissue color	Preconditioning	Ambient	**<0.001**	***	-9.88
			ST	**<0.001**	***	-8.06
			VT	**<0.001**	***	-4.54
M. digitata	ΔF/Fm'	Preconditioning	Ambient	**0.004**	**	-0.99
			ST	**0.003**	**	-1.95
			VT	**0.021**	*	-1.39
	Tissue color	Preconditioning	Ambient	**0.003**	**	-1.95
			ST	**0.021**	*	-1.25
			VT	**0.038**	*	-1.18
P. verrucosa	ΔF/Fm'	Preconditioning	Ambient	**<0.001**	***	-4.3
			ST	**<0.001**	***	-1.92
			VT	**<0.001**	***	-2.35
	Tissue colo	Preconditioning	Ambient	**<0.001**	***	-6.05
			ST	**<0.001**	***	-4.09
			VT	**<0.001**	***	-3.78
S. pistillata	ΔF/Fm'	Preconditioning	Ambient	**<0.001**	***	-17.08
			ST	**<0.001**	***	-4.05
			VT	**<0.001**	***	-4.9
	Tissue color	Preconditioning	Ambient	**<0.001**	***	-5.42
			ST	**<0.001**	***	-5.86
			VT	**<0.001**	***	-5.76

Table S6: Statistical comparison of coral receptiveness between preconditioning treatments based on paired delta values (Δ=post-heat minus post-preconditioning). Significant Bonferroni adjusted *p*-values (linear mixed effect model) are highlighted in bold.

Species	Analysis (Δ values)	Term	Contrast (Pairwais post-hoc)	*P value* (Adj. BH)	Signif.
G. fascicularis	ΔF/Fm'	Preconditioning	ΔAmbient - ΔST	**<0.01**	**
			ΔAmbient - ΔVT	**<0.001**	***
	Tissue color	Preconditioning	ΔAmbient - ΔST	**<0.001**	***
			ΔAmbient - ΔVT	**<0.001**	***
P. rus	ΔF/Fm'	Preconditioning	ΔAmbient - ΔST	**<0.01**	**
			ΔAmbient - ΔVT	**<0.001**	***
	Tissue color	Preconditioning	ΔAmbient - ΔST	0.0921	
			ΔAmbient - ΔVT	0.201	
A. muricata	ΔF/Fm'	Preconditioning	ΔAmbient - ΔST	**<0.001**	***
			ΔAmbient - ΔVT	**<0.001**	***
	Tissue color	Preconditioning	ΔAmbient - ΔST	**<0.001**	***
			ΔAmbient - ΔVT	**<0.001**	***
			ΔST - ΔVT	**<0.05**	*
M. digitata	ΔF/Fm'	Preconditioning	ΔAmbient - ΔST	1	
			ΔAmbient - ΔVT	0.968	
	Tissue color	Preconditioning	ΔAmbient - ΔST	**<0.05**	*
			ΔAmbient - ΔVT	**<0.01**	**
P. verrucosa	ΔF/Fm'	Preconditioning	ΔAmbient - ΔST	**<0.001**	***
			ΔAmbient - ΔVT	**<0.001**	***
	Tissue color	Preconditioning	ΔAmbient - ΔST	**<0.01**	**
			ΔAmbient - ΔVT	**<0.001**	***
S. pistillata	ΔF/Fm'	Preconditioning	ΔAmbient - ΔST	**<0.001**	***
			ΔAmbient - ΔVT	**<0.001**	***
	Tissue color	Preconditioning	ΔAmbient - ΔST	1	
			ΔAmbient - ΔVT	1	

Table S7: Statistical results of the recovery of preconditioned corals after 30 days. Recovery was estimated as the difference (effect size as hedges g) between the heat and control treatments within each treatment for each response variable. Significant *p*-values (two-tailed permutation t-test) are shown in bold. A non-significant *p*-value indicates that there is no statistically detectable difference between the physiological parameters of corals previously exposed to heat and those of the control group, suggesting that the corals have recovered. Missing measurements due to the death of all fragments are indicated by "NA".

Species	Analysis	Term	Contrast (heat - control)	Effect size (hedges_g)	***P value*** **(two-sided permutation t-test)**	Signif.
G. fascicularis	ΔF/Fm' (recovery)	Preconditioning	Ambient	-1.20	**0.028**	*
			ST	-2.55	**0.028**	*
			VT	-1.37	0.114	
	Tissue color (recovery)	Preconditioning	Ambient	-1.37	**0.028**	**
			ST	-0.95	0.2	
			VT	-0.98	0.114	
P. rus	ΔF/Fm' (recovery)	Preconditioning	Ambient	-1.20	**<0.001**	***
			ST	-2.55	**0.015**	*
			VT	-1.37	0.720	
	Tissue color (recovery)	Preconditioning	Ambient	-2.72	**<0.001**	***
			ST	-0.86	**0.05**	*
			VT	-0.70	0.065	
A. muricata	ΔF/Fm' (recovery)	Preconditioning	Ambient	NA	NA	
			ST	NA	NA	
			VT	NA	NA	
	Tissue color (recovery)	Preconditioning	Ambient	NA	NA	
			ST	NA	NA	
			VT	NA	NA	
M. digitata	ΔF/Fm' (recovery)	Preconditioning	Ambient	-0.51	0.486	
			ST	-0.08	0.885	
			VT	-0.91	0.114	
	Tissue color (recovery)	Preconditioning	Ambient	-0.37	0.342	
			ST	-0.28	1	
			VT	0.25	0.685	
P. verrucosa	ΔF/Fm' (recovery)	Preconditioning	Ambient	NA	NA	
			ST	NA	NA	
			VT	NA	NA	
	Tissue color (recovery)	Preconditioning	Ambient	NA	NA	
			ST	NA	NA	
			VT	NA	NA	
S. pistillata	ΔF/Fm' (recovery)	Preconditioning	Ambient	NA	NA	
			ST	NA	NA	
			VT	NA	NA	
	Tissue color (recovery)	Preconditioning	Ambient	NA	NA	
			ST	NA	NA	
			VT	NA	NA	

Supplementary Figures

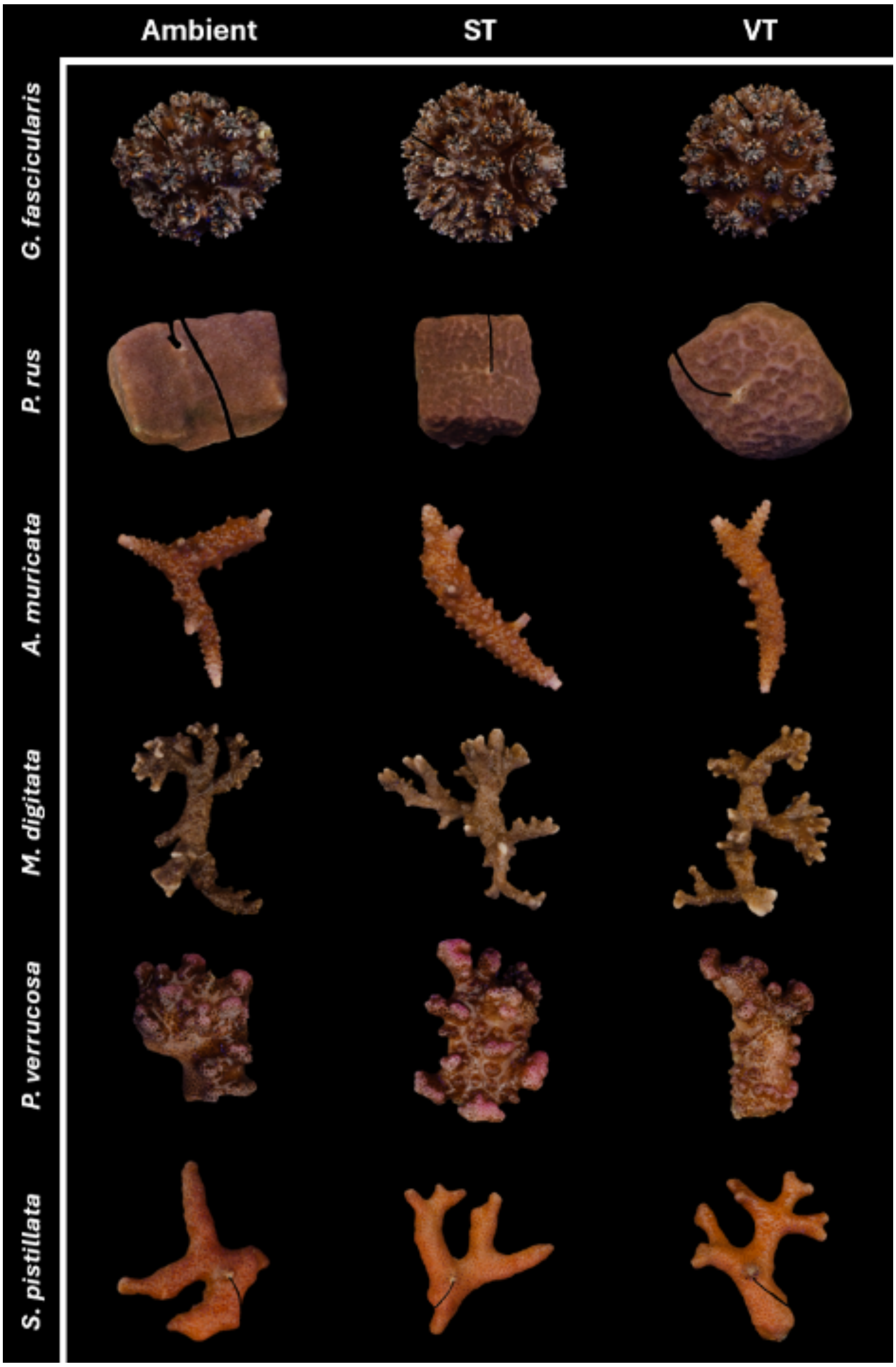

Figure S1: Tissue color changes after preconditioning (and before the heat stress assays). Comparison of coral tissue color between preconditioning treatment at the "post-preconditioning" time point. Some of the corals from ST and VT treatments were visibly and significantly (see Fig. 2, Tab. S2) paler than the Ambient as a result of the stress-hardening process.

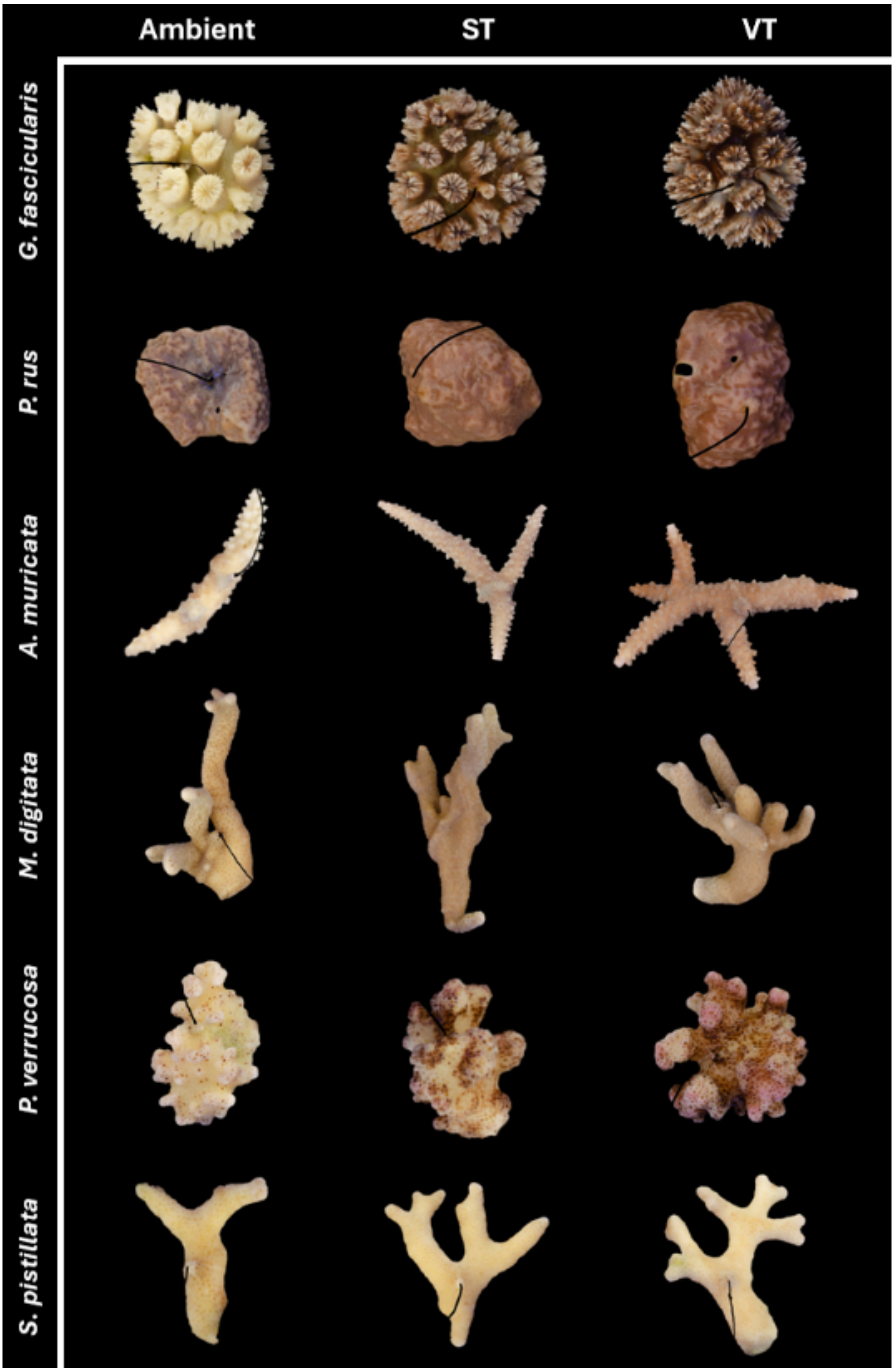

Figure S2: Tissue color changes after the heat stress assays. Comparison of coral tissue color between preconditioning treatment at the "post-heat" time point. Overall, corals from the Ambient treatment bleached more severely than ST and VT corals (see Fig. 3, Tab. S5, S6).

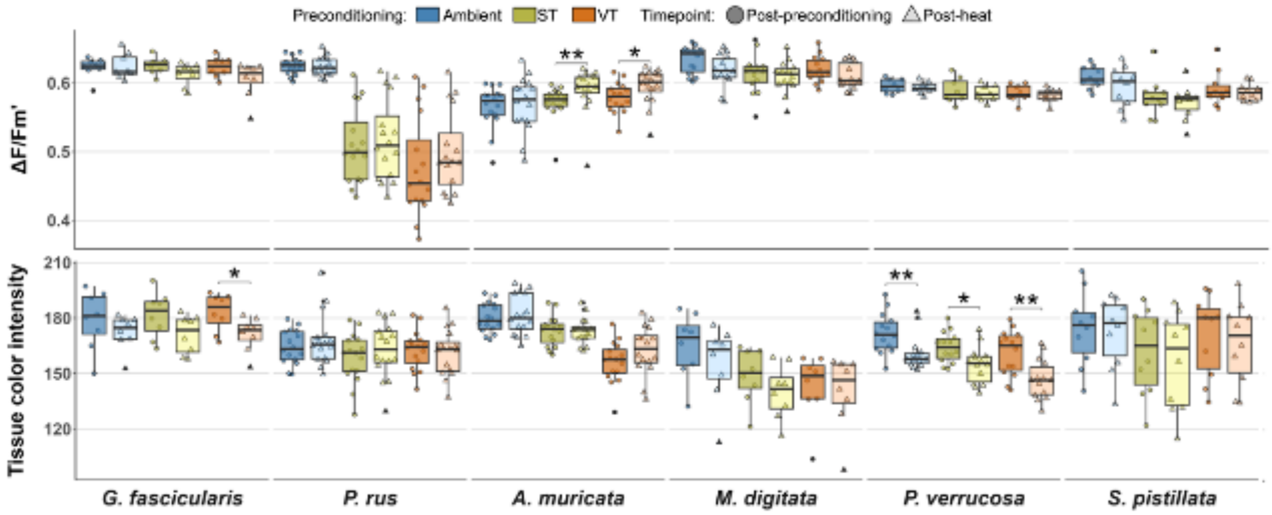

Figure S3: Corals physiological parameters in the Control treatment. The changes in effective quantum yield (ΔF/Fm') (A) and tissue color intensity (C) within each preconditioning group of the Control treatments are shown as boxplots, comparing paired post-heat (lighter color) to post-preconditioning (darker color) values. Data are displayed as boxplots with raw data points. Connecting lines between boxes indicate significant differences between time points ($p < 0.001$***, < 0.01**, < 0.05* from Kruskal-Wallis and post hoc Wilcoxon test).
